# Reconstructing Middle and Upper Paleolithic human mobility in Portuguese Estremadura through laser ablation strontium isotope analysis

**DOI:** 10.1073/pnas.2204501120

**Published:** 2023-05-08

**Authors:** Bethan Linscott, Alistair W. G. Pike, Diego E. Angelucci, Matthew J. Cooper, James S. Milton, Henrique Matias, João Zilhão

**Affiliations:** ^a^Oxford Radiocarbon Accelerator Unit, University of Oxford, Oxford OX1 3QY, United Kingdom; ^b^Department of Archaeology, University of Southampton, Southampton SO17 1BF, United Kingdom; ^c^Dipartimento di Lettere e Filosofia, Università degli Studi di Trento, Trento 38122, Italy; ^d^Centro de Arqueologia da Universidade de Lisboa, Faculdade le Letras, Universidade de Lisboa, 1600 - 214 Lisboa, Portugal; ^e^School of Ocean and Earth Science, National Oceanography Centre, University of Southampton, Southampton SO14 3ZH, United Kingdom; ^f^Instituci Catalana de Recerce i Estudis Avançats, Barcelona 08010, Spain; ^g^Department of History and Archaeology, University of Barcelona, Barcelona 08007, Spain

**Keywords:** Palaeolithic, isotopes, strontium, mobility, Portugal

## Abstract

Understanding mobility and landscape use is important in reconstructing subsistence behavior, range, and group size, and it may contribute to our understanding of phenomena such as the dynamics of biological and cultural interactions between distinct populations of Upper Pleistocene humans. However, studies using traditional strontium isotope analysis are generally limited to identifying locations of childhood residence or nonlocal individuals and lack the sampling resolution to detect movement over short timescales. Here, using an optimized methodology, we present highly spatially resolved ^87^Sr/^86^Sr measurements made by laser ablation multicollector inductively coupled plasma mass spectrometry along the growth axis of the enamel of two marine isotope stage 5b, Middle Paleolithic Neanderthal teeth (Gruta da Oliveira), a Tardiglacial, Late Magdalenian human tooth (Galeria da Cisterna), and associated contemporaneous fauna from the Almonda karst system, Torres Novas, Portugal. Strontium isotope mapping of the region shows extreme variation in ^87^Sr/^86^Sr, with values ranging from 0.7080 to 0.7160 over a distance of c. 50 km, allowing short-distance (and arguably short-duration) movement to be detected. We find that the early Middle Paleolithic individuals roamed across a subsistence territory of approximately 600 km^2^, while the Late Magdalenian individual parsimoniously fits a pattern of limited, probably seasonal movement along the right bank of the 20-km-long Almonda River valley, between mouth and spring, exploiting a smaller territory of approximately 300 km^2^. We argue that the differences in territory size are due to an increase in population density during the Late Upper Paleolithic.

## Significance

Laser ablation MC-ICP-MS allows in situ strontium isotope data to be obtained for incrementally formed bioapatites such as enamel with extremely high spatial resolution. Here, we provide a large-scale application of the method comparing the mobility and subsistence behavior of Middle and Upper Paleolithic humans in the same landscape. These remains and the fauna analyzed alongside come from the Almonda karst system (Portuguese Estremadura). Data suggest that regional Middle Paleolithic individuals roamed across a subsistence territory of approximately 600 km^2^, while Upper Paleolithic individuals moved seasonally and exploited a smaller territory of approximately 300 km^2^.

Understanding the mobility patterns of Middle and Upper Paleolithic human populations can aid in the reconstruction of their subsistence behavior, cognitive ability, geographical range, and group size. In particular, diachronic comparisons of landscape use and subsistence strategies of anatomically modern humans and Neanderthals may provide insights into the factors that led to the assimilation of the latter in Europe approximately 45,000 to 40,000 y ago (1). The Iberian Peninsula occupies a central position in debates concerning the interaction between these two human groups around the Middle–Upper Paleolithic transition (2–6), but direct isotopic studies of Middle and Upper Paleolithic human and animal mobility from this region have so far been limited.

Strontium isotope analysis of bulk enamel is a routine methodology for exploring past mobility (7), but until the application of laser ablation multicollector inductively coupled plasma mass spectrometry (LA-MC-ICP-MS) analysis (8–10), the sample size required precluded its application to intratooth Sr isotope analysis on human material. Now, the spatial resolution of optimized laser ablation MC-ICP-MS analysis allows >1,000 individual ^87^Sr/^86^Sr measurements along a typical human enamel sample (11). This method, when combined with sequential oxygen isotope analysis, can provide seasonal mobility data for humans and fauna in areas where geological variation is significant across short distances. By combining indirect evidence for settlement patterns and resource exploitation (such as faunal assemblages and lithics) with isotopic data, a more detailed picture of the subsistence behaviors of human groups can be developed.

The Pleistocene cave deposits of Portuguese Estremadura’s Central Limestone Massif offer an invaluable opportunity to carry out such a study *ceteris paribus* because of the region’s highly variable patchwork geology. Two localities therein, the Almonda karst system (Torres Novas) and the Gruta do Caldeirão (Tomar), provide one of the richest collections of Middle and Late Pleistocene human and animal remains in western Iberia, characterized by human remains dating to the Lower, Middle, and Upper Paleolithic (6, 12–15). Here, we present sequential, high-resolution strontium isotope data for the tooth enamel of three individuals from the Almonda karst system (two Neanderthals from Gruta da Oliveira and a Magdalenian human from Galeria da Cisterna) obtained through LA-MC-ICP-MS analysis along with sequential strontium and oxygen isotope data for contemporaneous fauna from the Almonda sites (*SI Appendix*, Table S1).

## Methodological Background

The ^87^Sr/^86^Sr ratio of bedrock is a function of the initial ^87^Rb content and time elapsed since its formation (7), and as such, rocks of different geological ages will exhibit distinct ^87^Sr/^86^Sr values. The process of weathering introduces strontium ions from the underlying geology into the biosphere—soils reflect the ^87^Sr/^86^Sr ratios of the bedrock from which they are derived, and groundwater echoes the strontium isotope signal of deposits through which it passes (16). Local soil and water ^87^Sr/^86^Sr ratios are subsequently adopted by the tissues of plants growing within a given catchment, and these values are passed along the food chain from producer to consumer with minimal fractionation (17, 18). Strontium readily substitutes for calcium in calcium-bearing minerals due to both cations sharing the same valency (19). The calcium component of the primary mineral phase present in animal bones and teeth, hydroxyapatite [Ca_10_(PO_4_)_6_(OH)_2_], is therefore commonly replaced by strontium during biomineralization (20). Strontium and calcium are metabolized by animals in the same way, and as such, teeth and bones will reflect the ^87^Sr/^86^Sr ratios of food and water consumed during the formation or remodeling of these respective tissues (19, 21). As such, the ^87^Sr/^86^Sr ratios observed in incrementally formed, biomineralized tissues of a given organism should primarily echo the isotopic signal of the underlying geology of the area in which said tissue was formed or remodeled, allowing mobility across geologies of varying ages to be detected through sequential analysis. For archaeological studies, the assumption is that baseline strontium isotopic values sampled today (e.g., from plants or sediments) reflect those of the past. Problems with this assumption, e.g., from changes in erosion patterns, can cause uncertainties in the interpretation of mobility.

The strontium isotopes in teeth will reflect the strontium-weighed mean of the dietary components, which need to be considered. While it is acknowledged that Neanderthals occupied a high trophic level, at least in the steppe-tundra regions of northwestern Europe (e.g., ref. 22), the biopurification of strontium with the trophic level means that meat is significantly depleted in strontium relative to plants. A study of modern dietary strontium (23) shows that nuts contain c. 74× the strontium of red meat, pulses (c. 20×), vegetables (c. 16×), and cereals and fruits (c. 9.5×). Furthermore, rabbit and poultry contain less strontium than red meat. It is likely, therefore, that our strontium isotope measurements in human enamel are strongly weighted to the locations of plant gathering rather than the migratory ranges of the animal species consumed, although we acknowledge that some uncertainty remains.

The oxygen isotope composition of precipitation differs as a result of variables including altitude, latitude, rainout relating to the distance from the coast, and air temperature (24). The latter of these parameters, air temperature, varies with the seasons in mid and high latitudes. The highest δ^18^O values correlate with the warmest seasonal temperatures, and the lowest δ^18^O values are associated with the coldest (25). Animal body water reflects the oxygen isotope composition of the meteoric water ingested directly or indirectly by the individual, and as such, those δ^18^O values are incorporated by mammalian tissues as they form (25). Seasonal temperature variations in rainfall δ^18^O are therefore recorded as sinusoidal profiles in incrementally formed tissues that develop over the course of a year or longer. Conversely, if an individual engages in migratory behavior, the measured δ^18^O profile may appear flat or dampened since the act of avoiding climatic extremes ensures a degree of stability in experienced environmental conditions (26).

Since mammalian tooth enamel forms incrementally during childhood and is not subsequently remodeled (20), it provides an invaluable time-resolved archive of biogeochemical information that can be accessed if sufficiently high sampling resolution can be achieved. The rate and geometry of the complex process of enamel mineralization in diphyodont mammals have, however, been a topic of debate with regard to its implications for the recovery of time-resolved isotope data (27). Recent work on modern caprine tooth enamel combining LA-MC-ICP-MS analysis and tracking via the global positioning system has nevertheless demonstrated that sequential ^87^Sr/^86^Sr can be used to reconstruct mobility with a monthly resolution, at least in sheep and ibex (28). While the mineralization rate of human enamel is less well understood, spatially resolved δ^18^O analyses of Neanderthal teeth from Payre, France (29), have demonstrated that seasonal variations in oxygen isotope values in individual teeth are consistent with well-documented modern human crown formation times (30). A recent study of ^87^Sr/^86^Sr profiles in modern and archaeological human tooth enamel has further demonstrated that chronologically sequenced ablation data are not the result of end-member mixing and can be considered reflective of transitions between bioavailable strontium sources (31). Here, we present mobility of fauna at the seasonal scale by anchoring ^87^Sr/^86^Sr data to δ^18^O where possible. Additional information on our approach and the methods used can be found in *SI Appendix*, Texts S1 and S2.

## Site Background

**Fig. 1.**
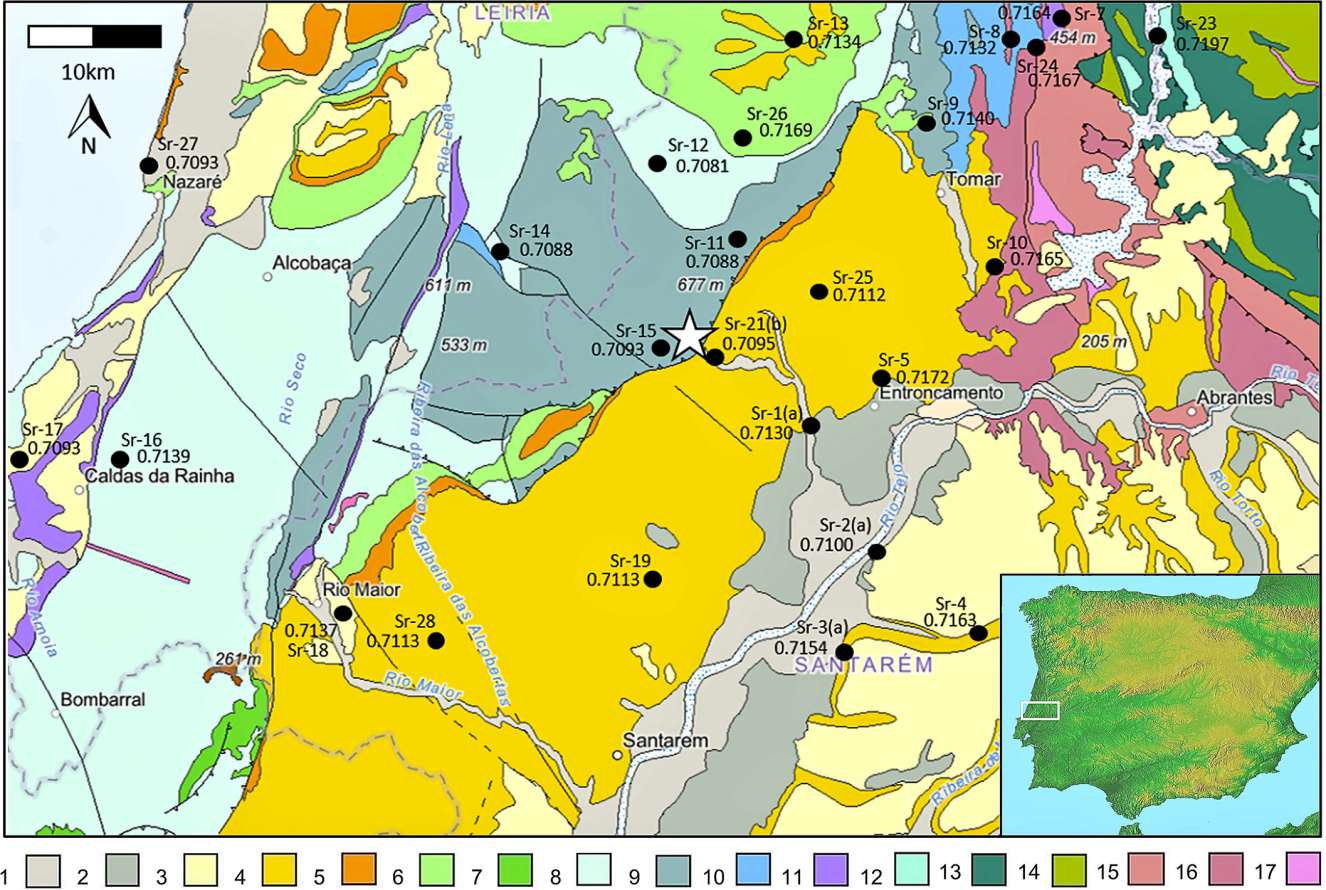
Geological map of the sampled area, whose position in Iberia is indicated by the rectangle in the *Inset*. The star denotes the Almonda spring. Sampling spots are indicated by their number and mean ^87^Sr/^86^Sr value (*SI Appendix*, Table S4). 1. Holocene sands, gravel, silts, and clays; 2. Pleistocene conglomerates, sandstones, siltstones, and clay stones; 3. Pliocene sandstones, siltstones, and clay stones; 4. Miocene sandstones, clay stones, conglomerates, and limestones; 5. Paleogene sandstones, conglomerates, clay stones, and siltstones; 6. Upper Cretaceous limestones, sandstones, marl, and dolomites; 7. Lower Cretaceous sandstones, limestones, marl, and dolomites; 8. Upper Jurassic limestones, marl, and sandstones; 9. Middle Jurassic limestones, marl, and dolomites; 10. Lower Jurassic limestones, marl, and dolomites; 11. Triassic sandstones, clay stones, and evaporites; 12. Silurian phyllites, shales, and metacherts; 13. Ordovician quartzites, quartzophyllites, and metaconglomerates; 14. Cambrian schists, gneisses, migmatites, and amphibolites; 15. Neoproterozoic phyllites, metacherts, metaconglomerates, and shales; 16. Neoproterozoic schists, gneisses, metacherts, migmatites, and amphibolites; and 17. deformed orthogneisses, granites, and diorites. After ref. 32.

The Almonda karst system (Fig. 1 and *SI Appendix*, Figs. S1 and S2) is located approximately 100 km to the northeast of Lisbon, at the southern edge of the Mesozoic Central Limestone Massif of Portuguese Estremadura. It consists of around 12 km of mapped subterranean galleries in association with the spring of the Almonda River, which are accessed by multiple entrances—fossil outlets—located at successive elevations up a limestone escarpment (*SI Appendix*, Figs. S3 and S4). The latter is part of the ~40-km-long, NE–SW-oriented tectonic fault separating the massif’s mountains and plateaus from the Cenozoic alluvial plain of the Tagus River.

The Gruta da Oliveira is a collapsed entrance to the system located approximately 40 m above the river’s spring. The site contains a 6-m-thick Middle Paleolithic sequence and has yielded a total of nine Neanderthal skeletal elements (13), three of which are teeth (cf. *SI Appendix*, Figs. S5 and S6). One of these teeth was recovered from layer 17, and the other two from layer 22, which is dated to 109.3 ± 28.4 ka by single grain optically stimulated luminescence and assigned an age of 93,250 ± 3,450 y via Bayesian modeling of the stratigraphic succession’s chronological span; the latter places layers 15 to 25, whose charcoal assemblage is dominated by the cold-adapted Scots pine (*Pinus sylvestris*), in marine isotope stage-5b (MIS-5b) (33). The presence of characteristic Levallois stone tool technology, an abundance of hearth material, and tens of thousands of faunal remains (many of them burnt) show that the site was used for the processing and consumption of locally hunted fauna including rhino, horse, red deer, ibex, and tortoise (34, 35).

The Galeria da Cisterna, which measures 100 m in length, opens approximately 5 m above the extant spring and 35 m below the Gruta da Oliveira. Underlying a Holocene dark cave earth deposit, it contains cemented sediment remnants of Last Glacial Maximum and Tardiglacial age. Level 3 of the AMD1 locus, a Magdalenian deposit dated on bone samples to 10,820 ± 60 BP (GrA-9722; 12,708 to 12,882 cal BP) and 11,755 ± 80 BP (OxA-11129; 13,476 to 13,785 cal BP; *SI Appendix*, Tables S2 and S3), yielded fifteen human bone and dental specimens. Based on the characteristics of the dental remains (cf. *SI Appendix*, Fig. S7), it is suggested that a minimum of three individuals of varying ages (young child to young adult) are represented. The small faunal assemblage comprises red deer, Iberian ibex, and wild boar along with smaller mammals such as rabbit and hare (13, 36, 37).

The Lapa dos Coelhos is a small cavity situated approximately 10 m above the Galeria da Cisterna (*SI Appendix*, Figs. S3 and S4) containing two well-preserved Magdalenian layers, 4 and 3, dated to, respectively, 12,240 ± 60 BP (GrA-18377; on *P. sylvestris* charcoal) and 11,660 ± 60 BP (GrA-18376; on a red deer bone), i.e., to between 13,353 and 14,803 cal BP (*SI Appendix*, Tables S2 and S3). A substantial faunal assemblage was recovered from both layers, comprising primarily rabbits, red deer, Iberian ibex, and fish, mostly freshwater cyprinids (e.g., *Barbus* sp.) but including *Salmo* sp. and *Alosa* sp. Analysis of the Magdalenian lithic assemblages and the presence of bone artifacts interpreted as fishhooks in layer 4 has led to the interpretation of the site as a temporary one, occupied intermittently for the exploitation of specific (and perhaps seasonal) resources (36–39).

## Results

The analysis of sediment leachates from 27 locations within the study area demonstrates a high degree of geographical variation in ^87^Sr/^86^Sr values (Fig. 1 and *SI Appendix*, Table S4). These range between 0.7081 (Sr-12) and 0.7362 (Sr-22), with less radiogenic values common along the Jurassic limestone massif and more radiogenic values evident in the Neogene sedimentary basin of the Tagus River and the Paleozoic phyllites to the northeast of the Almonda karst system. Our values are consistent with James et al.’s (40) large-scale strontium isotope baseline map of Portugal who give Sr isotope values in the range of 0.7082 to 0.7112 for the area incorporating the Central Limestone Massif, the Neogene sedimentary basin, and the Quaternary deposits associated with the Tagus River.

**Fig. 2.**
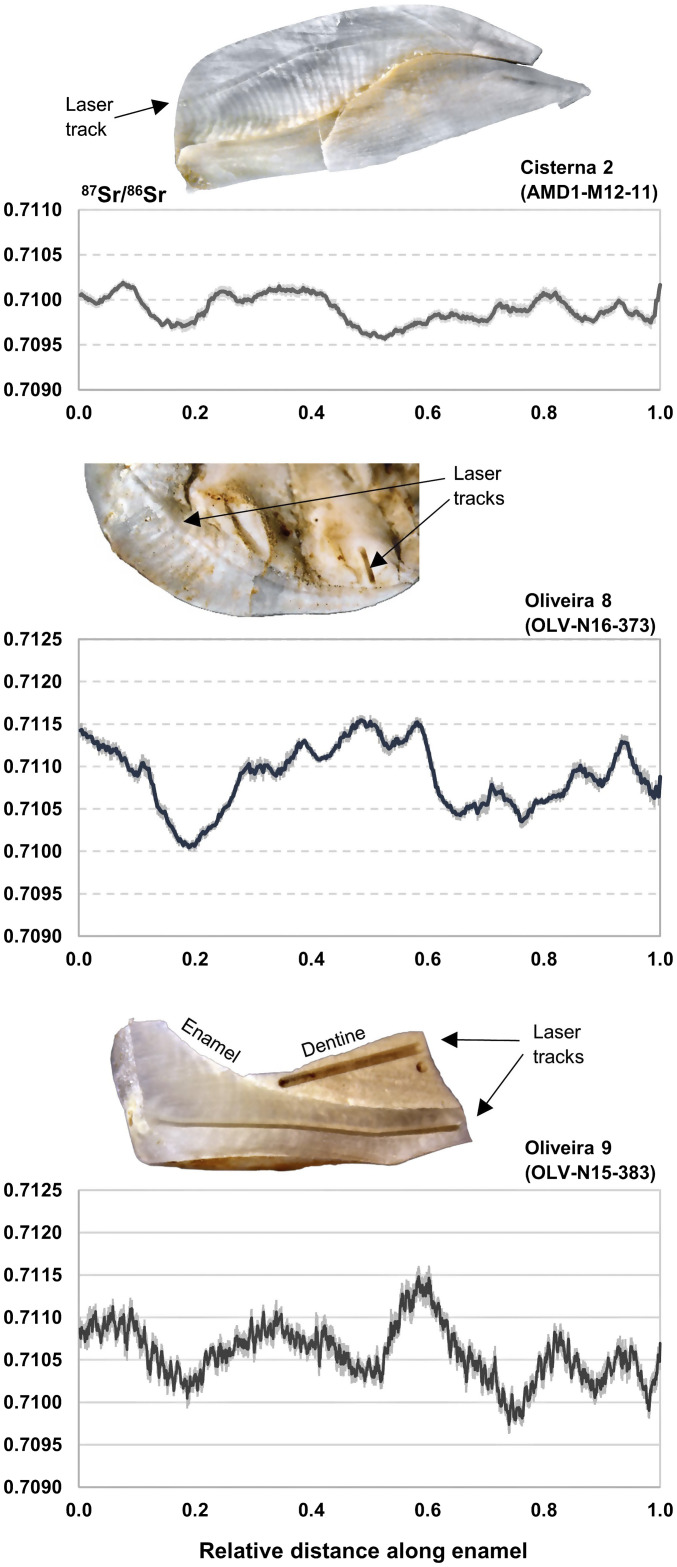
Sequential ^87^Sr/^86^Sr enamel profiles, obtained through LA-MC-ICP-MS analysis, of the three human teeth analyzed, plotted from *Left* (top of crown) to *Right* (enamel cervix), following the growth axis.

The sequential strontium isotope data obtained for the two Neanderthal teeth from layer 22 (Oliveira 8 and Oliveira 9; *SI Appendix*, Figs. S5 and S6) are presented in Fig. 2. Both individuals exhibit inhomogeneous strontium isotope profiles, with ^87^Sr/^86^Sr oscillating between minimum and maximum values of approximately 0.7100 and 0.7115, respectively. Between four and five different strontium isotope catchments can be observed in each profile, which can be accounted for within the range of bioavailable strontium isotope values in the study area.

A selection of Mousterian fauna (*SI Appendix*, Table S1) from different units of the Gruta da Oliveira MIS-5b sequence comprising horse (n = 3), ibex (n = 2), red deer (n = 1), and *Stephanorhinus sp*. (n = 2) was also analyzed, and the data are presented in *SI Appendix*, Fig. S8. All three horse molars exhibit relatively homogeneous ^87^Sr/^86^Sr profiles with values ranging between 0.7095 and 0.7105, while the ibex ^87^Sr/^86^Sr profiles oscillate between less radiogenic values of approximately 0.7086 and 0.7095. The red deer ^87^Sr/^86^Sr values range between 0.7090 and 07095, while the two *Stephanorhinus sp.* individuals exhibit more radiogenic values ranging between 0.7100 and 0.7115. Accompanying oxygen isotope data obtained for two of the horse specimens, one ibex, one *Stephanorhinus* sp. individual, and the red deer specimen are plotted alongside the strontium isotope data.

The strontium isotope data obtained for the Magdalenian human premolar from Galeria da Cisterna (Cisterna 2; *SI Appendix*, Fig. S7) are presented in Fig. 2. Repeat oscillations between distinct values of approximately 0.7095 and 0.7100 can be observed in the ^87^Sr/^86^Sr profile. To demonstrate its primary position, the tooth was also directly dated (to 11,122 ± 34 BP; OxA-41,483; 12,926 to 13,107 cal BP) (36, 37) (*SI Appendix*, Tables S2 and S3).

An ibex maxilla (F3-88) and a red deer mandible (F3-72) from Magdalenian layer 4 of Lapa dos Coelhos were selected to undergo analysis alongside the Cisterna 2 human tooth. To demonstrate its primary position, the red deer mandible was directly dated (to 12,130 ± 43 BP; OxA-41484; 13,811 to 14,115 cal BP) (36, 37) (*SI Appendix*, Tables S2 and S3). The strontium and oxygen isotopes obtained are presented in *SI Appendix*, Fig. S9. The ibex individual exhibits molar strontium isotope profiles similar to those of the two Mousterian ibex, with values ranging between approximately 0.7086 and 0.7097. Two different isotope catchments can be observed in the red deer molars, with values of 0.7103 and 0.7113.

## Discussion

### Middle Paleolithic.

The strontium isotope profiles of the two Neanderthals (Oliveira 8 and Oliveira 9) suggest that these individuals engaged in systematic movement between four geological catchments during M_3_ and P_3_ crown formation, respectively [between 8.5 to 14.5 y and 3.5 to 6.5 y (30)]. The most radiogenic values observed in the enamel ^87^Sr/^86^Sr profiles of both individuals, approximately 0.7112 to 0.7115, are consistent with the Neogene sedimentary basin that lies between the Central Limestone Massif and the Tagus River (Sr-19, Sr-25, and Sr-28). The least radiogenic value observed, approximately 0.7100, is consistent with strontium isotope values observed in sediment leachates from Quaternary sedimentary deposits along the banks of the Tagus River (Sr-2a). Based on the wider bioavailable strontium isotope mapping of the study region, these values can be accounted for within a <20 km radius of the Almonda karst system.

**Fig. 3.**
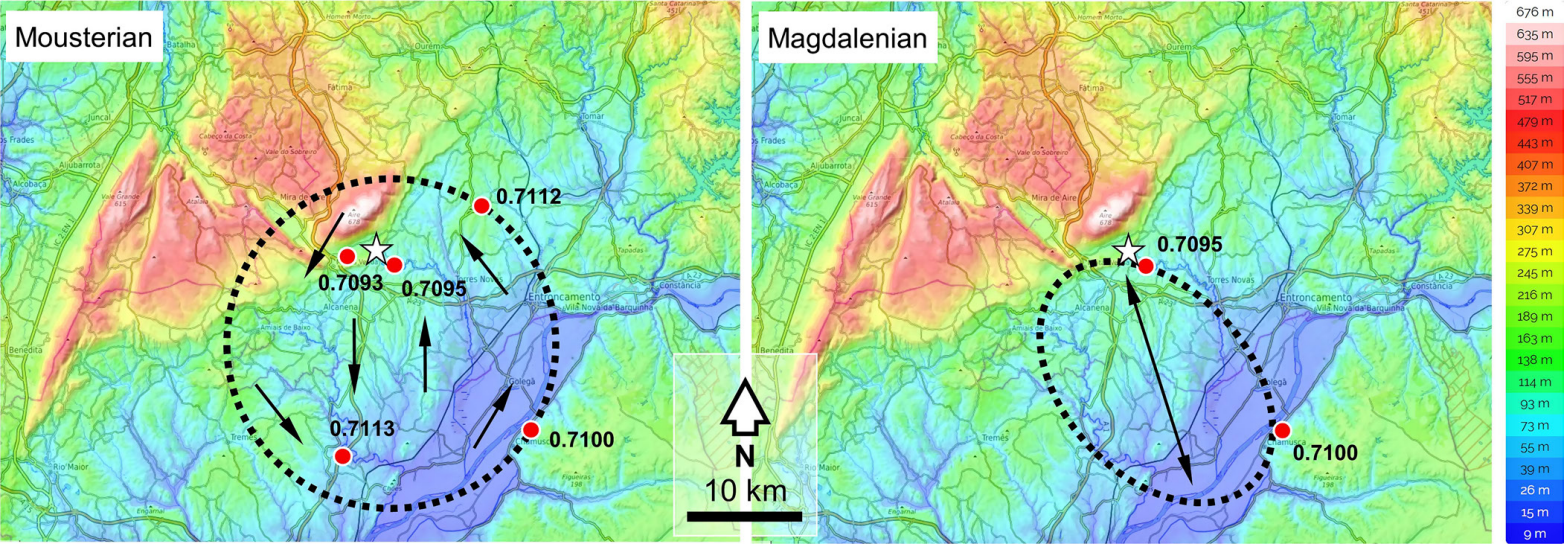
Modeled territories used by the Oliveira and Cisterna individuals. The star denotes the Almonda spring. The red dots denote the sampling localities within the delimited areas where soil leachates yielded Sr values consistent with the human values. The circular area modeled for the Mousterian (~600 km^2^) is bounded by points Sr-25 (0.7112) and Sr-2a (0.7100) consistent, and Sr-5 (0.7172), inconsistent, with the human tooth values. The elliptical area modeled for the Magdalenian (~300 km^2^) is bounded by points Sr-21b (0.7095) and Sr-2a (0.7100) consistent, and Sr-1a (0.7130) and Sr-19 (0.7113), inconsistent, with the human tooth values. Through the time span of their enamel formation, the two Mousterian individuals roamed across the Tagus basin and ventured into the Massif, while the Magdalenian individual moved regularly across a territory half the size that would not seem to have included the Massif and corresponds to terrain along the right bank of River Almonda. Given the predominantly plant source of dietary strontium, our results do not rule out hunting forays outside these ranges, but we have also shown that important prey species are present within these ranges for at least part or all of the year.

To the north, northeast, and east, however, ^87^Sr/^86^Sr ratios are consistently above 0.7115 beyond a distance of 10 to 15 km from the site. We estimate the settlement–subsistence territory as the largest circular or subcircular areas that satisfy the three conditions of a) containing the site, b) containing terrain with soil Sr values consistent with those measured on the human teeth, and c) containing no terrain with soil Sr values inconsistent with those measured on the human teeth (Fig. 3). This is c. 600 km^2^, mostly comprising lowland alluvial terrain in the Cenozoic basin of the Tagus and including the southern footslopes of the Serra d’Aire (Fig. 3); this evidence suggests that to the northeast and east, the two individuals moved within an area delimited by the drainage basin of the Atalaia stream (*SI Appendix*, Fig. S2). These inferences are consistent with the ranges suggested by studies of lithic raw material sourcing in the Mousterian deposits of Gruta da Oliveira (41).

**Fig. 4.**
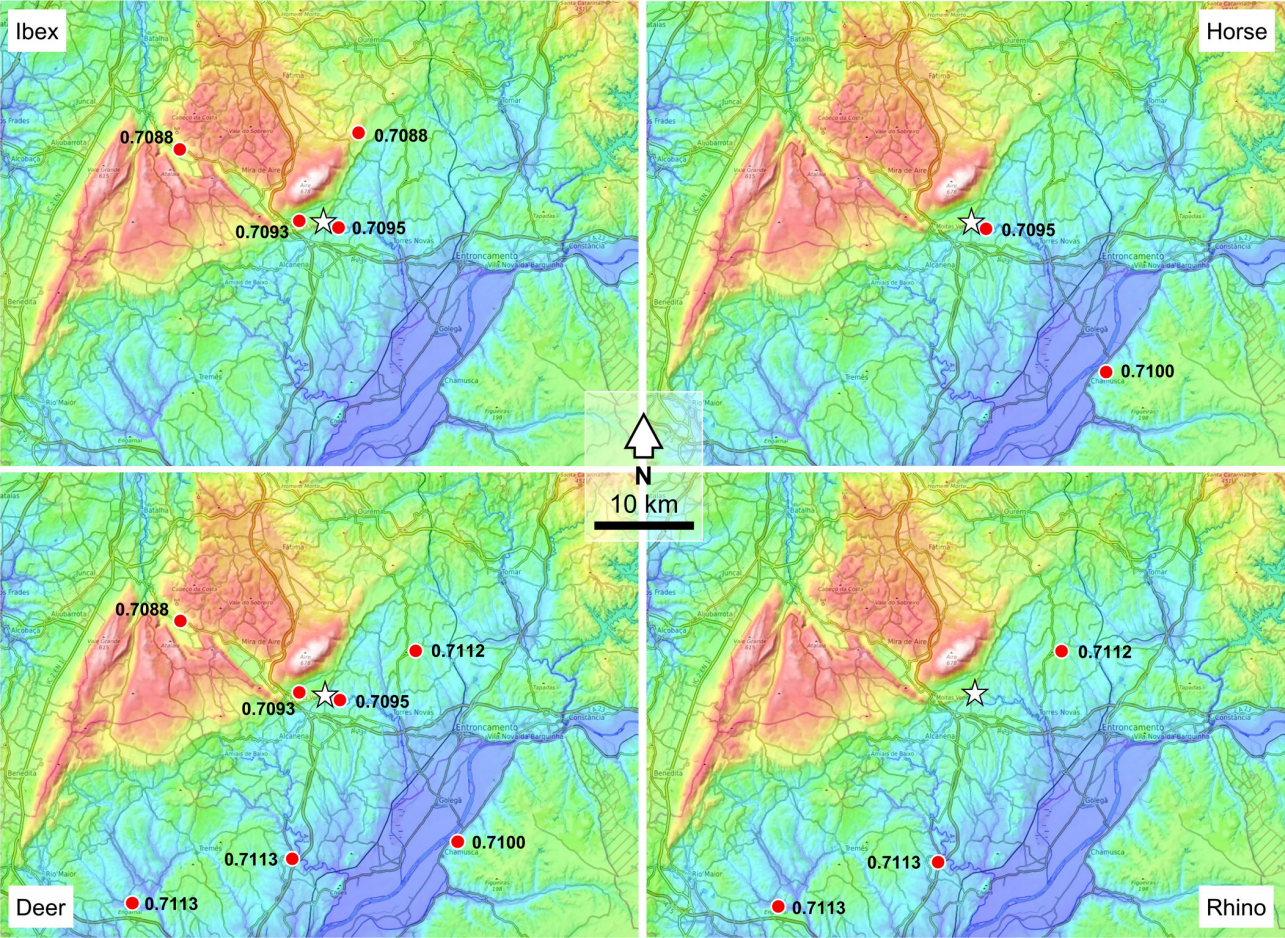
Potential ranges of the main Middle and Upper Paleolithic animal prey species represented in the Almonda karst sites, as indicated, for each taxon, by the sampling localities (red dots) where soil leachates yielded Sr values (indicated) within the range of the tooth values. The star denotes the Almonda spring. Elevations are color-coded as in Fig. 3.

Sequential strontium and oxygen isotope data for Middle Paleolithic fauna show horse, red deer, and extinct rhinoceros were present in the two Neanderthals’ subsistence territory all year round, and the Iberian ibex intersects with this when they move to lower altitudes in the summer. Horses, on the basis of their relatively homogeneous enamel ^87^Sr/^86^Sr profiles [*SI Appendix*, Fig. S8; representing 2 to 3 y of age (42)], likely occupied the alluvial plain along the right bank of the Tagus River throughout the year (Sr-2a; Figs. 1 and 4). Seasonal variation is not readily apparent in their δ^18^O profiles, which likely reflects their behavior as obligate drinkers (43) relying on the waters of the Tagus and Almonda rivers, where seasonal δ^18^O variation in local precipitation may be dampened due to long residence times (43, 44). Iberian ibex, on the other hand, are a mountainous species highly adapted to life among steep, rocky slopes. The 2^nd^ and 3^rd^ molars of our individuals likely represent formation times of around 12 mo based on modern sheep (45) and exhibit ^87^Sr/^86^Sr and δ^18^O values that reflect seasonal movement between higher altitudes in the Serra d’Aire and adjacent plateaus of the Central Limestone Massif (Sr-11 and Sr-14; Fig. 4 and *SI Appendix*, Fig. S8) and the lower altitudes at the source of the Almonda River (Sr-21b). This seasonal movement is similar to that observed in modern populations (46), although their high-altitude winter, low-altitude summer movements are the reverse of modern populations elsewhere in Iberia. However, given that the Serra d’Aire has a maximum altitude of c. 680 m, their movement is probably driven by the availability of food rather than extreme winter cold.

On the basis of the consistency of their ^87^Sr/^86^Sr profiles with sediment leachate values Sr-19, Sr-25, and Sr-28, the *Stephanorhinus sp.* individuals likely occupied the Neogene Tagus basin (Fig. 4 and *SI Appendix*, Fig. S8), subsisting upon varying proportions of browse and grass depending on quality and availability (47). Minimal intratooth variation in δ^18^O of approximately 2‰ is evident but does not reflect seasonal signals, suggesting that these individuals likely obtained the majority of their water from a source with a long oxygen residence time. The intratooth variation in ^87^Sr/^86^Sr for both individuals may reflect a degree of movement between the banks of the Tagus River and the Neogene basin. Similar small-scale seasonal mobility in *Stephanorhinus kirchbergensis* has been observed in isotope data from an individual from western Poland, where it is inferred that the individual spent the autumn months in more forested areas before occupying more open areas in winter, spring, and summer (48). The ^87^Sr/^86^Sr profiles of the single Middle Paleolithic red deer specimen are similar to those of the sediment leachates from the vicinity of the Almonda karst system (Sr-15, Sr-21b) and the Alvados polje (Sr-14) (Fig. 4 and *SI Appendix*, Figs. S2 and S8). Little variation can be observed along the growth axes of either tooth, suggesting that the individual likely subsisted entirely on the same geology during their formation. The δ^18^O data for both teeth show an approximately 1‰ increase in values from the crown toward the root, implying that the time period represented by these teeth was likely during the transition from colder to warmer months.

### Upper Paleolithic.

The sequential ^87^Sr/^86^Sr data for the Magdalenian individual from Galeria da Cisterna repeatedly oscillate between two geologically distinct regions, consistent with sediment values from the source of the Almonda River (Sr-21b) and the banks of the Tagus River (Sr-2a), of which the Almonda is a tributary. The ranging distance of the Magdalenian individual during the period of dental formation therefore falls within approximately 20 km of the Galeria da Cisterna and could well represent back-and-forth movement along the right bank of the river valley (Figs. 2 and 3) between spring (at the Almonda escarpment) and mouth (at the confluence with the Tagus). The size of the corresponding subsistence territory, estimated as above, is ~300 km^2^. This is in line with observations of raw material procurement in the contemporaneous Magdalenian layers of the adjacent Lapa dos Coelhos site; ~80% of which comes from sources in the intermediate Neogene terrain (38).

Like the Mousterian ibex specimens from Gruta da Oliveira, the strontium and oxygen isotope data suggest that the Magdalenian ibex individual from Lapa dos Coelhos (F3-88) engaged in some form of seasonal altitudinal mobility (Fig. 4 and *SI Appendix*, Fig. S9) between higher altitudes on the Serra d’Aire (Sr-14, Sr-11) in winter and lower altitudes in the vicinity of the Almonda karst system in summer (Sr-15; Sr-21b), where it intersects with the proposed subsistence territory for the Magdalenian individual. The intratooth δ^18^O variation is approximately 4‰, although in this relatively low-altitude terrain, the greater extent of oxygen isotope variation when compared to the Mousterian ibex may suggest more seasonal extremes in temperature during the Tardiglacial than during MIS-5b. The strontium isotope profiles obtained for the M1, M2, and M3 of the Magdalenian red deer from Lapa dos Coelhos (F3-72) are consistent with mobility between two distinct geologies (Fig. 4 and *SI Appendix*, Fig. S9). The least radiogenic values fall within those of sediment leachates from the banks of the Tagus River (Sr-2a), within the proposed subsistence territory of the human individual and correspond to the warmer months. The highest are consistent with areas of the Neogene sediments of the Tagus basin (Sr-19, Sr-28) that may lie outside the subsistence territory. δ^18^O values are similar to the ibex, with intratooth variation approximately 4‰, and with the highest oxygen isotope values corresponding to the least radiogenic ^87^Sr/^86^Sr troughs and the lowest oxygen isotope values corresponding to the most radiogenic ^87^Sr/^86^Sr peaks. This may reflect a degree of local seasonal mobility, perhaps in response to the seasonal availability of good forage.

Both the red deer and ibex are present for at least part of the year in the subsistence territory proposed for the Magdalenian individual, with their strontium isotope values overlapping with the two strontium isotope catchments identified in the human tooth (Figs. 3 and 4). The cyclical movement of Cisterna 2 between two different geological catchments is suggestive of seasonal movement and did not include the limestone mountains and plateaus extending to the north and northwest of the Almonda escarpment. The presence of fishhooks and fish vertebra and direct evidence for the consumption of aquatic resources provided by δ^13^C and δ^15^N (−18.65**‰** and 10.89**‰**, respectively, *SI Appendix*, Fig. S11) indicate the importance of freshwater aquatic resources and a small marine input likely from the anadromous fish (*Salmo* sp. and *Alosa* sp.) represented in the bone assemblage (38). The movement between riparian locations on the Almonda and Tagus rivers might be linked to the importance of these resources.

The continuity between the Middle and the late Upper Paleolithic in the presence and location of some of the prey species suggests that the marked differences in human movement and subsistence territory between the two periods are unlikely to have been determined by changes in ecology. This is supported by paleobotanical evidence. *P. sylvestris* is the dominant taxon in the charcoal assemblage from the MIS-5b levels of Gruta da Oliveira and is also well represented in the Upper Magdalenian of the region, namely in layer 4 of Lapa dos Coelhos and in coeval layer F of Lapa do Picareiro, 5 km to the north across the Serra d’Aire (39, 49, 50). Nowadays restricted to the high mountains of Portugal, above 1,500 m, this taxon denotes the altitudinal compression of vegetation belts under colder climatic conditions and is a proxy for an open landscape of Scots pine and heathland across the lowlands of Portuguese Estremadura (49, 51).

Against a comparable landscape setting, horses, well represented in Solutrean layer 8 of Lapa dos Coelhos, are absent from the Almonda karst’s Upper Magdalenian faunal assemblages (Galeria da Cisterna and layers 3 to 4 of Lapa dos Coelhos) (14, 38). A similar trend is apparent at Lapa do Picareiro, where horses are present through Gravettian levels T-Z but absent from Upper Magdalenian level F/G, whose abundant faunal remains are of rabbit (97%), red deer (2%), and wild boar (1%) and include a few hundred fish bones of the same taxa represented at Lapa dos Coelhos (52–54). A reduction in subsistence territory in the Magdalenian might therefore explain the absence of horses in the faunal assemblage. Horses were extensively hunted in preceding periods and persisted in the region until the early-mid-Holocene (55) but are not present in the Galeria da Cisterna and Lapa dos Coelhos assemblages. Either the Magdalenians’ restricted territory did not overlap with horses or horses were butchered and consumed away from the Almonda caves.

Consideration of exchange networks as revealed by the occurrence of shell ornaments in the Upper Paleolithic of central Portugal is also consistent with a marked contraction of territories in the Tardiglacial. In the Upper Magdalenian, such ornaments are almost entirely of freshwater taxa. In the Solutrean and the Gravettian, despite a much lower sea level making for a significantly greater geographical separation between littoral and inland groups, shell beads are overwhelmingly of marine taxa (*SI Appendix*, Text S5).

The abundance of fish and rabbit remains in the regional Upper Magdalenian suggests that people were moving down the food chain to accommodate a decrease in territory size, presumably as a consequence of growth in population numbers and population density. A similar argument has been put forth based on the subsistence importance acquired in the Tardiglacial (for the first time in the Paleolithic of Europe) by the regular exploitation of freshwater fish apparent in broadly coeval French and Italian sites (56–58). The increase in archaeological site frequency per millennium observed through the Middle and the Upper Paleolithic of northern Spain and SW France is consistent with these inferences (59, 60).

The subsistence territory estimated for the Upper Paleolithic human from Galeria da Cisterna is about half the area of that proposed for the two Neanderthals from Gruta da Oliveira. It represents the opposite trend to the more than two-fold increase in home range between the Middle Paleolithic and Late Upper Paleolithic estimated for SW France and the northern European plain based on raw material sourcing (61). This contrast is to be expected, given the lower latitude of central Portugal and the littoral location of the study area, which is far beyond the Eurasian steppe-tundra domain. This geography would also have made for the impact of climate oscillations to be lesser and for the carrying capacity of the land to be up to one order of magnitude greater than in European regions to the north of the Pyrenees—for the 13- to 15-ka cal BP interval, climate envelope modeling estimates are that central Portugal could have supported population densities of up to 16 to 23 people/100 km^2^ (62). Based on these estimates, the model Upper Magdalenian territory derived from the strontium evidence could have harbored up to 50 to 70 people and so would have been big enough to sustain a hunter-gatherer band of typical size.

## Conclusions

Here, we present highly spatially resolved sequential strontium isotope data for two Middle Paleolithic humans and one Upper Paleolithic human from the Almonda karst system in Portuguese Estremadura. Sequential strontium and oxygen isotope analysis of Middle and Upper Paleolithic fauna suggests that all four species sampled (ibex, red deer, horse, and rhino) were either resident or seasonally available within a short distance from the Almonda karst sites. Data for Middle Paleolithic horses and red deer suggest little to no mobility during enamel formation, while Middle Paleolithic rhino engaged in small-scale, local mobility that may have been seasonal in nature. Upper Paleolithic red deer were also seasonally mobile across the study area during dental formation, and both Middle and Upper Paleolithic ibex engaged in altitudinal mobility.

Based on ^87^Sr/^86^Sr mapping of the study area, we conclude that the Neanderthal individuals occupied a subsistence territory of ~600 km^2^, and likely subsisted on four geological catchments that were visited and revisited within a 10 to 15 km radius of the Almonda spring, consistent with a settlement–subsistence system leaning toward Binford’s “forager” model of mobility (63) and with suggestions that Neanderthals may have had high basal metabolic rates, requiring them to make frequent but short-distance forages (64). In contrast, the Magdalenian individual subsisted primarily upon resources obtained, probably seasonally, from two geological catchments along a 20-km stretch of the right bank of the Almonda River, between the spring and the mouth, representing a subsistence territory of ~300 km^2^. While we recognize that a switch in diet in the Magdalenian to high-strontium local resources (e.g., fish or freshwater mollusks) may mask the true extent of the subsistence territory, we see no evidence in the enamel for covariance of Sr isotopic values with Sr concentrations, which we would predict if this was the case (*SI Appendix*).

The reduction in territory size inferred from our sequential strontium isotope data may be related to increased population density during the late Upper Paleolithic. Future in situ oxygen isotope analysis of the human tooth enamel, e.g., ref. 65, may enable us to better understand the seasonal timescales involved in this process.

## Supplementary Material

Appendix 01 (PDF)

## Data Availability

The dataset for this study is available as an Excel Workbook (66).
