# Supplementary material for: Reconstructing Middle and Upper Paleolithic human mobility in Portuguese Estremadura through laser ablation strontium isotope analysis
Source: Proc Natl Acad Sci U S A. 2023 May 8;120(20):e2204501120. doi: 10.1073/pnas.2204501120 (PMC10193924; doi:10.1073/pnas.2204501120)
Supplement: Supplementary file 1 — Appendix 01 (PDF) [file pnas.2204501120.sapp.pdf]

Supplementary Information for

Reconstructing Middle and Upper Palaeolithic human mobility in Portuguese Estremadura through laser ablation strontium isotope analysis

Bethan Linscott*, Alistair W.G. Pike*, Diego E. Angelucci, Matthew J. Cooper, J Andy Milton, Henrique Matias, João Zilhão

**Corresponding authors*

Email: bethan.linscott@arch.ox.ac.uk, a.w.pike@soton.ac.uk

This PDF file includes:

SI Text

SI References

Figures S1 to S12

Tables S1 to S5

Supplementary Text

S1. Materials and Methods

Human and animal teeth were sampled in the stable isotope laboratory at the Department of Archaeology, University of Southampton. Enamel slices approximately 1-2mm thick were removed from human and animal tooth specimens parallel to the growth axis of the tooth using a diamond-tipped cutting disc and a Dremel tool, subsequently ultrasonicated in Milli-Q water, and dried in a vacuum oven. To minimize the impact of sampling, sections of human enamel that had been exposed through damage during excavation were targeted where possible. Because of their archaeological value and the need to retrieve them post-analysis, human tooth samples were mounted in a blu-tack block for ablation. Slices of enamel were removed from animal tooth crowns in the same manner, but were instead mounted in Epoxy resin and polished using 220, 800 and 1200 grit papers on a Buehler Metaserv Grinder- Polisher. The three human teeth were microCT-scanned prior to sampling in order to mitigate against the inflicted damage.

Tooth enamel slices were analysed using two LA-MC-ICP-MS systems at the School of Ocean and Earth Science ICP-MS Research Facility at the University of Southampton; a New Wave Research UP193FX excimer laser coupled to a Thermo Finnegan Neptune MC-ICP-MS and an Elemental Scientific Lasers NWR193 excimer laser coupled to a Thermo Scientific Neptune Plus MC-ICP-MS. Identical analytical protocols were used on both systems. Following loading, the sample cell holder was purged with He for 10 minutes. Ablated material was transported to the MC-ICP-MS using a He carrier gas mixed after the sample cell with Ar and N_2 via a quartz mixing bulb. The LA-MC-ICP-MS was tuned to minimize oxide production. This was monitored via $^{254}(UO)+/^{238}U+$ following the method of de Jong (1). Samples of NIST 610 glass were ablated along continuous 100μm lines with a translation rate of 5μm/s-1 and a repetition rate of 10Hz. The instrument was tuned through the adjustment of a number of parameters including gas flow rates and the Z position of the plasma torch, until the $^{254}(UO)+/^{238}U+$ was minimised. This is regarded as a proxy for the production efficiency of calcium phosphates in the plasma, with conditions producing a ratio of less than 0.01% deemed to be suitable for the subsequent analysis of enamel unknowns. $^{87}Sr/^{86}Sr$ data were collected with an integration time of 1.049 seconds per measurement cycle. An on-peak blank was collected prior to each analysis. The $^{87}Sr/^{86}Sr$ value was corrected for the ^{87}Rb interference using the natural $^{87}Rb/^{85}Rb$ ratio of 0.385617. Instrumental mass fractionation was corrected for using an exponential fractionation law (2) and the natural $^{86}Sr/^{88}Sr$ ratio of 0.1194 (3).

Over the course of these analyses, two known-value bioapatite standards were repeatedly analysed. An ashed and pressed bovine bone pellet known as BP (produced at the University of Bristol as part of a previous study; [1]) was utilised, along with several isotopically homogenous pig teeth produced during a feeding study at the University of Bristol (4). Across analysis days, repeated measurement of BP (n=29) gave a mean offset and standard error of 124 ± 95 ppm over the reference TIMS value, whilst the repeat analysis of the enamel from PT 232 (n=32) and PT 238 (n=78) gave a mean offset and standard error of 65 ± 60 and 103 ± 31 ppm over the TIMS values, respectively.

Prior to isotopic analysis, human enamel samples were surface cleaned by rapidly ablating the plotted laser path to remove any surface contaminants. For this purpose, a spot size of 150μm, a repetition rate of 10Hz and a 50μm/s^{-1} translation rate was utilised for human teeth. For surface cleaning of the animal teeth, which are generally larger, the translation rate was increased to 100μm/s^{-1}. For human enamel samples, the subsequent analysis along the pre-ablated laser track used a 150μm spot size, a repetition rate of 20Hz and a translation rate of 2μm/s^{-1}; for fauna the translation rate was increased, depending on the specimen, to between 30μm/s^{-1} and 50μm/s^{-1} to account for the greater length of the samples. Gas blank data were collected for 60 seconds prior to each analysis with the same parameters, except with

the laser shutter closed. ^{89}Y was monitored as a proxy for rare earth elements (REEs) which can be taken up diagenetically (5) and present numerous isobaric interferences in the mass range of strontium. In one sample (N15-17), the data was trimmed in a region where higher levels of ^{89}Y covaried with $^{87}Sr/^{86}Sr$.

Sediment samples were collected at locations representative of the different geological and soil types within a *c.* 50km radius of the sites. Subsoil samples were taken away from locations of obvious agriculture. Soils and sediments were leached for two weeks in RO 18Ω water at room temperature. The leach solution was filtered and divided into aliquots for the measurement of strontium concentration using a ThermoFisher Scientific XSeries2 ICP-MS, and for isotopic analysis using a ThermoFisher Scientific Triton Plus TIMS. Both aliquots were processed on ~50ml Sr-Spec resin (Triskem, France) columns using 2ml of 3M sub-boiled nitric acid to elute and the strontium collected in 1.5ml of MQ water. The samples were dried and loaded in 1.5ml of sub-boiled 1M hydrochloric acid onto an outgassed tantalum filament using a tantalum activator solution for subsequent TIMS analysis, using a multi dynamic procedure with an ^{88}Sr beam of 2V. Fractionation was corrected using an exponential correction normalized to $^{86}Sr/^{87}Sr$ = 0.1194.

For oxygen isotope analysis, a second enamel section parallel to the first was taken from the tooth crown. Surface dirt and any dentine was removed using a dental bur. The sections were then subdivided into sequential samples, which measured between 2-5mm long depending on the length of the tooth. Each of these sub-samples was ground into a powder using an agate pestle and mortar, and subjected to a 0.1M acetic acid wash for 15 minutes in order to remove exogenous carbonates. Each sample was then washed five times in ultrapure Milli-Q water and freeze-dried to return them to a powdered state.

Analysis was carried out at the Stable Isotope Mass Spectrometry Laboratory at the National Oceanography Centre, Southampton. Approximately 500µg of each sample was transferred to a Thermo KEIL IV carbonate device and automatically reacted with 106.7% phosphoric acid at 90°C in order to evolve CO2. The CO2 was then dried and cryogenically transferred to a Thermo Finnegan MAT 253 isotope ratio mass spectrometer. The oxygen isotope data are presented as δ-values in permil (‰) relative to the international standard Vienna Standard Mean Ocean Water (VSMOW). The typical measurement uncertainty, based on the repeated analysis of international and in-house standards, is 0.2‰.

S2. Consideration of diagenetic uptake of strontium

The uptake of Sr by teeth has the potential to alter or overprint the biogenic signal, confounding the interpretation of movement from Sr isotope values. While it has been observed that dentine and bone are generally very susceptible to Sr uptake, enamel is usually considered more immune because of its lower porosity and lower organic content (e.g. (6)). However, although diagenetic Sr signals in tooth enamel are rarely reported in the literature, the vast majority of archaeological Sr isotope studies are on fauna and humans from later prehistory to the recent past (e.g. (7)), and there are insufficient studies from older periods to assess the susceptibility of tooth enamel to Sr uptake over longer periods of time.

Estimates of the rate of uptake of Sr by tooth enamel from faunal teeth in contexts inundated by the North Sea (i.e. waterlogged conditions with known diagenetic Sr isotope ratio) suggest that biogenic Sr signals in enamel can become overprinted in 15-150 ka (8), although these timescales will be longer in drier conditions where the burial matrix pores and tooth enamel pores will be less coupled. Nevertheless, one should exercise caution when assuming measured Sr isotopic values are biogenic in enamel older than a few tens of thousands of years.

We compare distributions of Sr isotopic values between the enamel and dentine as a check for diagenetic Sr uptake in one of the oldest teeth in this study (OLV9, *c.* 93 ka; Fig. S10). The similarities in the variation of Sr isotopes between the enamel and the dentine show that even the more susceptible dentine has not suffered significant diagenetic Sr uptake. The effect of such uptake on the dentine (assuming it is uniform) would be to move the apparent Sr isotopic values towards the diagenetic value and to reduce the magnitude of the variation of the Sr isotopes in the measured profile. If the enamel were also affected by Sr uptake, it is likely to be less affected than the dentine (Lewis estimates 100-fold less, based on porosity differences (8)), and the apparent Sr isotope values of the enamel and dentine would diverge. It is significant therefore that the Sr isotope values in the enamel and dentine are broadly similar, both have a similar variation, and neither overlap with the likely diagenetic Sr isotope ratio, assumed to reflect Mesozoic limestone, i.e. in the range of 0.7068 -0.7077 (9). We therefore conclude that since the dentine has not been significantly affected by Sr uptake then we can discount Sr uptake in the enamel.

To have a biogenic, or near-biogenic Sr isotopic signal preserved in dentine is exceptional. Sr isotopic measurements on dentine (or bone) are usually made as part of the process of investigating the 'local' isotopic range for Sr, on the assumption that uptake will mean that the Sr values will reflect the Sr in the immediate burial environment rather than anything biogenic. There are cases where suites of teeth from the same context have been used to show partial overprinting of the biogenic signal in dentine — e.g. ((10) Fig. 5) and (11) — but there are very few cases reported where the dentine is not affected by significant Sr uptake. In Oliveira, It is likely that the carbonate cementation of some of the cave sediments, and the formation of carbonate crusts (12) limited the water percolation through the deposits containing the Neanderthal teeth. This would reduce the coupling of the pore water in the burial matrix with the pores of the enamel and dentine, limiting or halting the diffusion of Sr into the teeth.

S3. Sediment sampling rationale

Sediment samples were collected at locations representative of the different geological and soil types within a *c.* 50 km radius of the sites, away from locations of obvious agriculture. The field trips to acquire the samples took place on July 14-15, 2014. Selection of sampling locations aimed at characterizing how soils' Sr content varied as a function of the different combinations of bedrock and soil profiles present in the region.

In terms of rock types and ages, the geology of this sector of the western Iberian Peninsula is very diverse and includes Proterozoic and Paleozoic metamorphic rocks belonging to the geological basement, Paleozoic igneous lithotypes, Mesozoic sedimentary rocks (often carbonate ones), as well as Cenozoic and Quaternary sedimentary rocks and sediments (mostly terrigenous). Geological data were gathered from the different sheets of the Geological Map of Portugal (1:50,000 and 1:100,000), available on the Geoportal of LNEG (*Laboratório Nacional de Energia e Geologia*; https://geoportal.lneg.pt/mapa/).

Due to Pleistocene uplifting and subsequent erosion (e.g. (15)), the soil profiles of this sector of Portugal are often derived from Tardiglacial or Holocene soil formation. Ancient soils are preserved only within karst features or structural depressions. For this reason, soil properties are often strongly controlled by the physico-chemical characteristics of their parent material. Most soil samples were collected from cambisols, fluvisols or regosols, and only a few specimens come from podzols or luvisols. Soil data were taken from (16).

S4. Stable isotope analysis

Stable carbon ($^{13}C/^{12}C$) and nitrogen ($^{15}N/^{14}N$) isotope analysis of collagen was carried out in order to directly investigate the diet of the Upper Palaeolithic individual from Galeria da Cisterna. Collagen preservation within the Almonda karst system is highly variable, limiting the range of comparative faunal data available with which to generate an isotopic baseline. A total of three Upper Palaeolithic red deer from Lapa dos Coelhos are available. Collagen was extracted from bone (fauna) and dentine (human) samples following a modified Longin (17) method at the University of Southampton.

Aliquots of collagen extracted at the University of Southampton were run in duplicate using a Thermo EA1110 elemental analyser coupled to a dual-pumped Sercon 20-20 stable isotope mass spectrometer (OEA Laboratories). Results were corrected to certified standards ($\delta^{13}C$; vPDB; $\delta^{15}N$; AIR) and are presented in Fig S11 and Table S5 .

The average $\delta^{13}C$ and $\delta^{15}N$ values for the Lapa dos Coelhos red deer are -20.22 ± 0.8‰ and 3.82 ± 0.45‰ respectively; consistent with terrestrial C_3 herbivores. The Upper Palaeolithic human from Galeria da Cisterna exhibits a collagen $\delta^{13}C$ value of -18.65‰ and a $\delta^{15}N$ value of 10.89‰. The latter is 7.07‰ more enriched than the average $\delta^{15}N$ values of the red deer; two trophic positions higher based on an assumed ^{15}N trophic enrichment factor of 3.4‰ (18). This suggests that species occupying higher trophic positions likely contributed to the dietary protein intake of the individual. Given the presence of contemporaneous anadromous (*Salmo sp.* and *Alosa sp.*) and freshwater (*Cyprinidae sp.* and *Barbus sp.*) fish remains (along with fishhooks) in the deposits of Lapa dos Coelhos, it is likely that aquatic protein contributed to the elevated $\delta^{15}N$ value. The vertebrae of four fish specimens from Lapa dos Coelhos were selected for $\delta^{13}C$ and $\delta^{15}N$ analysis, but unfortunately no collagen could be recovered.

S5. Consideration of dietary strontium concentration

It is important to note that strontium isotope profiles may be skewed towards the $^{87}Sr/^{86}Sr$ values of resources with higher strontium concentrations. Vegetables and fish, for example, contain an order of magnitude more strontium per kilogram than red meat (19). Certain nuts that were likely available to Middle and Upper Palaeolithic humans can exhibit even greater strontium concentrations than meat, vegetables, and fish. Molluscs in particular have been found to hyperaccumulate strontium by three orders of magnitude in freshwater communities (20, 21). These variations in strontium concentration can complicate the interpretation of strontium isotope profiles in tooth enamel — for example, in a diet made up predominantly of meat, it is feasible that the majority of the strontium could be derived from plant foods (22). Likewise, an omnivorous diet may be swamped by strontium derived from a comparatively small contribution of marine molluscs.

The carbon and nitrogen isotope values of the Galeria da Cisterna human, combined with the presence of the remains of freshwater and anadromous fish (along with fishing implements) at Lapa dos Coelhos suggest that these freshwater resources did contribute to the diet of the Upper Palaeolithic individual, and therefore the enamel $^{87}Sr/^{86}Sr$ values may be weighted towards those resources. In theory, we therefore have to consider the possibility that the Galeria da Cisterna individual ranged further than the banks of the Tagus, consuming foods from the more radiogenic catchments beyond the modeled subsistence range, and that these more radiogenic values may have been overpowered by the $^{87}Sr/^{86}Sr$ values of the fish.

However, if such were the case, we would expect a correlation between the Sr concentration and isotope values, where peaks in Sr should be associated with the tending of the $^{87}Sr/^{86}Sr$ towards the values of the high strontium foods (0.7092 in the case of anadromous fish and 0.7100 in the case of freshwater molluscs). We see no obvious covariance in the ^{88}Sr and $^{87}Sr/^{86}Sr$ (Fig. S12) for the Magdalenian individual.

Furthermore, marine mollusc food taxa were found neither at Lapa dos Coelhos (the mollusc remains retrieved in layer 3 are all ornamental species, namely, >30 specimens of *Theodoxus fluviatilis*, a freshwater taxon, 15 of which perforated, and two specimens of the marine taxon *Littorina obtusata*, both perforated) nor at Galeria da Cisterna (those retrieved in layer 3, in association with the human remains, are also perforated ornamental taxa, namely, three *Theodoxus fluviatilis* and one *Hynia reticulata*) (23-24). The testimonial presence of marine taxa in these bead assemblages needs not imply that Upper Magdalenian Almondans regularly (e.g., seasonally) visited the coast, which is >50 km away (as the crow flies) across the limestone massif (plateau of Santo António and Candeeiros mountain range; Fig. S2), and would have been even more distant in the Tardiglacial, given the extent of the submerged continental platform (*c.* 40 km). Such a presence is parsimoniously explained via exchange and in any case contrasts markedly with the pattern displayed by the shell bead assemblage from Gruta do Caldeirão, 25 km north-east of the Almonda spring (25-26). Indeed, despite the greater distance from the seaside, only two of the 46 perforated shells retrieved in the Solutrean and Early Upper Palaeolithic levels of Gruta do Caldeirão are of freshwater species (*Theodoxus fluviatilis* and *Unio pictorum*); the other 44 are of marine taxa (*Antalis vulgare*, *Aporrhais pespelecani*, *Littorina obtusata*, and *Semicassis saburon*), and food taxa are entirely absent. The ornamental shell data are therefore entirely consistent with the notion that, for the people living to the east of the Central Limestone Massif, temporary residence in the Atlantic coast and subsistence exploitation of its resources (namely, shellfish) were off-limits. If anything, the bead data would in fact suggest that, by comparison with Last Glacial Maximum times, contact and exchange with littoral groups decreased markedly in the Tardiglacial, even as separation by distance decreased as a result of the rise in sea level. This pattern is in good agreement with a demographic interpretation of the strontium data.

To conclude: The $^{87}Sr/^{86}Sr$-derived home ranges of the ibex and red deer (which would have provided much greater dietary biomass but less strontium), the consistency of the Sr-modeled human range with raw material procurement, and the lack of archaeological evidence for the exploitation of marine fauna lead us to the parsimonious explanation that the majority of the nutritional resources consumed by the Galeria da Cisterna Magdalenian individual were available and likely collected between the spring and mouth of the Almonda River.

References

1. H. De Jong, Subsistence plasticity: A strontium isotope perspective on subsistence through intra-tooth enamel and inter-site variation by LA-MC-ICPMS and TIMS. PhD, University of Bristol (2013).
2. W. A. Russell, D. A. Papanastassiou, T. A. Tombrello, Ca isotope fractionation on the Earth and other solar system materials. *Geochimica et Cosmochimica Acta* **42**, 1075-1090 (1978).
3. A. O. Nier, The isotopic constitution of strontium, barium, bismuth, thallium and mercury. *Physical Review* **54**, 275 (1938).

4. J. Lewis, A. W. G. Pike, C. D. Coath, R. P. Evershed, Strontium concentration, radiogenic (87Sr/86Sr) and stable (δ88Sr) strontium isotope systematics in a controlled feeding study. *STAR: Science & Technology of Archaeological Research* **3**, 45-57 (2017).
5. Woodhead, S. Swearer, J. Hergt, R. Maas. In situ Sr-isotope analysis of carbonates by LA-MC-ICP-MS: interference corrections, high spatial resolution and an example from otolith studies. *Journal of Analytical Atomic Spectrometry* **20**, 22-27 (2005).
6. K. A. Hoppe, P. L. Koch, T. T. Furutani, Assessing the preservation of biogenic strontium in fossil bones and tooth enamel. *International Journal of Osteoarchaeology* **13**, 20-28 (2003).
7. J. A. Evans, C. A. Chenery, J. Montgomery, A summary of strontium and oxygen isotope variation in archaeological human tooth enamel excavated from Britain. *Journal of Analytical Atomic Spectrometry* **27**, 754-764 (2012).
8. J. Lewis, Lying through your teeth: strontium diagenesis in archaeological enamel. PhD, University of Bristol (2015).
9. W. H. Burke *et al.*, Variation of seawater $^{87}Sr/^{86}Sr$ throughout Phanerozoic time. *Geology* **10**, 516-519 (1982).
10. T. Reitmaier *et al.*, Alpine cattle management during the Bronze age at Ramosch-Mottata, Switzerland. *Quaternary International* **484**, 19-31 (2018).
11. P. Budd, J. Montgomery, B. Barreiro, R. G. Thomas, Differential diagenesis of strontium in archaeological human dental tissues. *Applied geochemistry* **15**, 687-694 (2000).
12. D. E. Angelucci, J. Zilhão, Stratigraphy and formation processes of the Upper Pleistocene deposit at Gruta da Oliveira, Almonda karstic system, Torres Novas, Portugal. *Geoarchaeology* **24**, 277-310 (2009).
13. P. J. Reimer *et al.*, The IntCal20 Northern Hemisphere radiocarbon age calibration curve (0–55 cal kBP). *Radiocarbon* **62**, 725-757 (2020).
14. F. Brock, T. Higham, P. Ditchfield, C. B. Ramsey, Current pretreatment methods for AMS radiocarbon dating at the Oxford Radiocarbon Accelerator Unit (ORAU). *Radiocarbon* ***52,*** 103-112 (2010).
15. J. Cabral, *Neotectónica em Portugal continental* (Instituto Geológico e Mineiro, Lisboa, 1995).
16. J. C. Cardoso, M. T. Bessa, M. O. B. Marado (1978) Portugal. Atlas do ambiente. Carta dos solos. (Comissão Nacional do Ambiente, Lisboa).
17. R. Longin. New method of collagen extraction for radiocarbon dating. *Nature* **230**, 241-24 (1971).
18. M. Minagawa, E. Wada, E. Stepwise enrichment of 15N along food chains: further evidence and the relation between δ15N and animal age. *Geochimica et Cosmochimica Acta* **48**(5), 1135-1140 (1984).
19. D. González-Weller, D. et al., Dietary intake of barium, bismuth, chromium, lithium, and strontium in a Spanish population (Canary Islands, Spain). *Food and Chemical Toxicology* **62**, 856-868 (2013).
20. I. L. Ophel. The fate of radiostrontium in a freshwater community. In (V. Schultz & A. W. Klement, Eds.) *Radioecology*. London: Chapmanand Hall, 213-216. (1963).
21. M. J. Schoeninger and C. S. Peebles. Effect of mollusc eating on human bone strontium levels. *Journal of Archaeological Science* **8**(4), 391-397 (1981).
22. C. M. Haverkort, V. I. Bazaliiskii and N. A. Savel'ev. Identifying hunter-gatherer mobility patterns using strontium isotopes. *Prehistoric hunter-gatherers of the Baikal region, Siberia: bioarchaeological studies of past life ways*. University of Pennsylvania Press, 217-238 (2011).

23. F. Almeida, D. E. Angelucci, C. Gameiro, J. Correia, T. Pereira, Novos dados para o Paleolítico Superior final da Estremadura Portuguesa: resultados preliminares dos trabalhos arqueológicos de 1997-2003 na Lapa dos Coelhos (Casais Martanes, Torres Novas). *Promontoria* **2**, 157-192 (2004).
24. E. Trinkaus, S. Bailey, S. J. M. Davis, J. Zilhão, Magdalenian Human Remains from the Galeria da Cisterna (Almonda karstic system, Torres Novas, Portugal). *O Arqueólogo Português* **V-1** 395-413 (2011).
25. F.-X. Chauvière, Industries et parures sur matières dures animales du Paléolithique supérieur de la grotte de Caldeirão (Tomar, Portugal). *Revista Portuguesa de Arqueologia* **5**, 5-28 (2002).
26. J. Zilhão *et al.*, Revisiting the Middle and Upper Palaeolithic archaeology of Gruta do Caldeirão (Tomar, Portugal). *PLoS One* **16**, e0259089 (2021).

Supplementary Figures

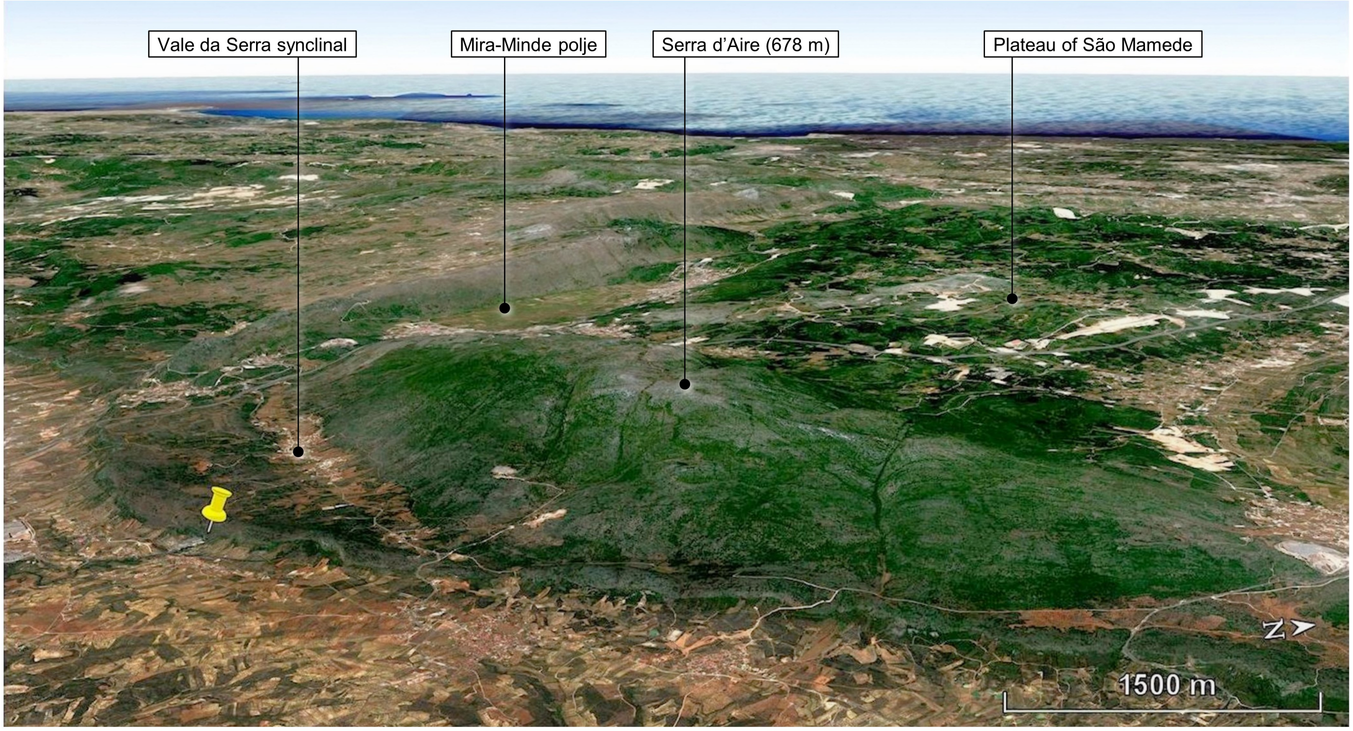

Fig. S1. GoogleEarth view (2009-12-31; 1.5x elevation) of the Central Limestone Massif of Portuguese Estremadura. The yellow pin indicates the position of the Almonda spring.

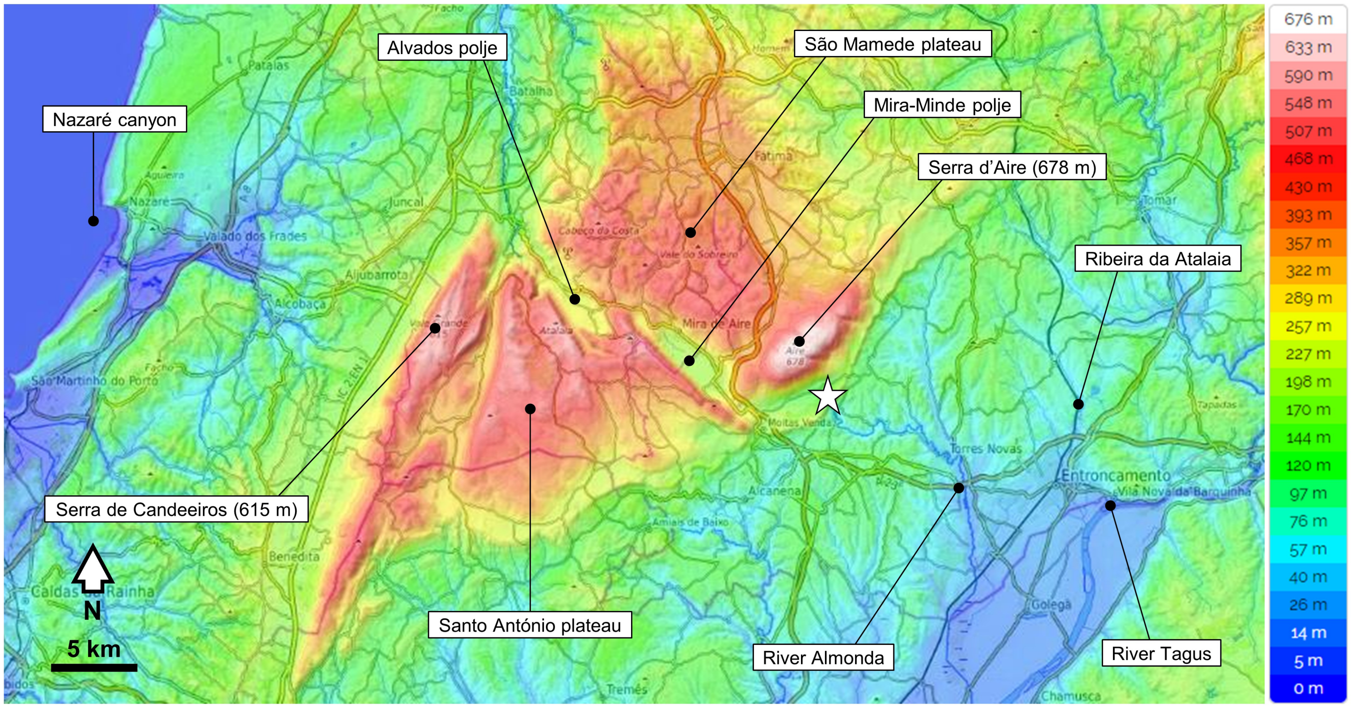

Fig. S2. Physical map of the Central Limestone Massif of Portuguese Estremadura and surrounding areas. The region's major geographic features are indicated. The star denotes the Almonda karst spring.

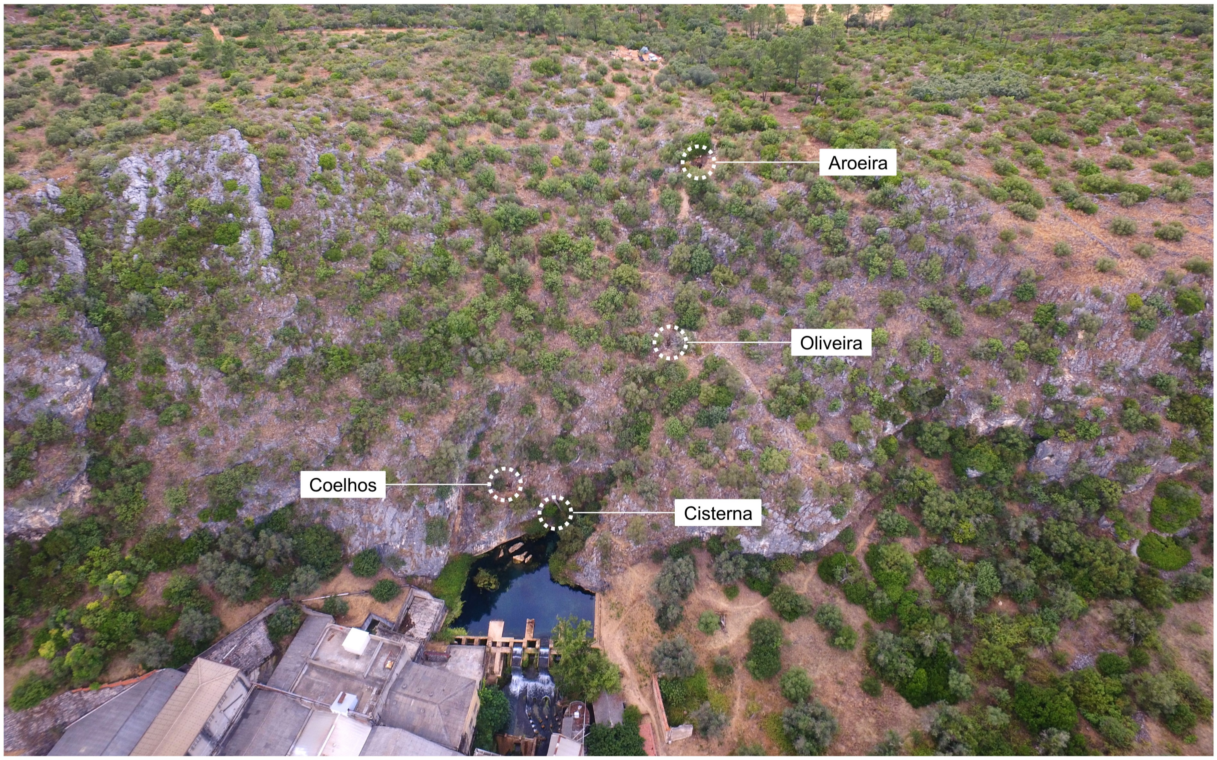

Fig. S3. Drone view of the Almonda spring (2016; courtesy Pedro Souto). The entrances to the different archaeological sites known in the associated karst system are indicated (Aroeira – Acheulean; Oliveira – Mousterian; Coelhos – Upper Paleolithic; Cisterna – Neolithic and Upper Paleolithic).

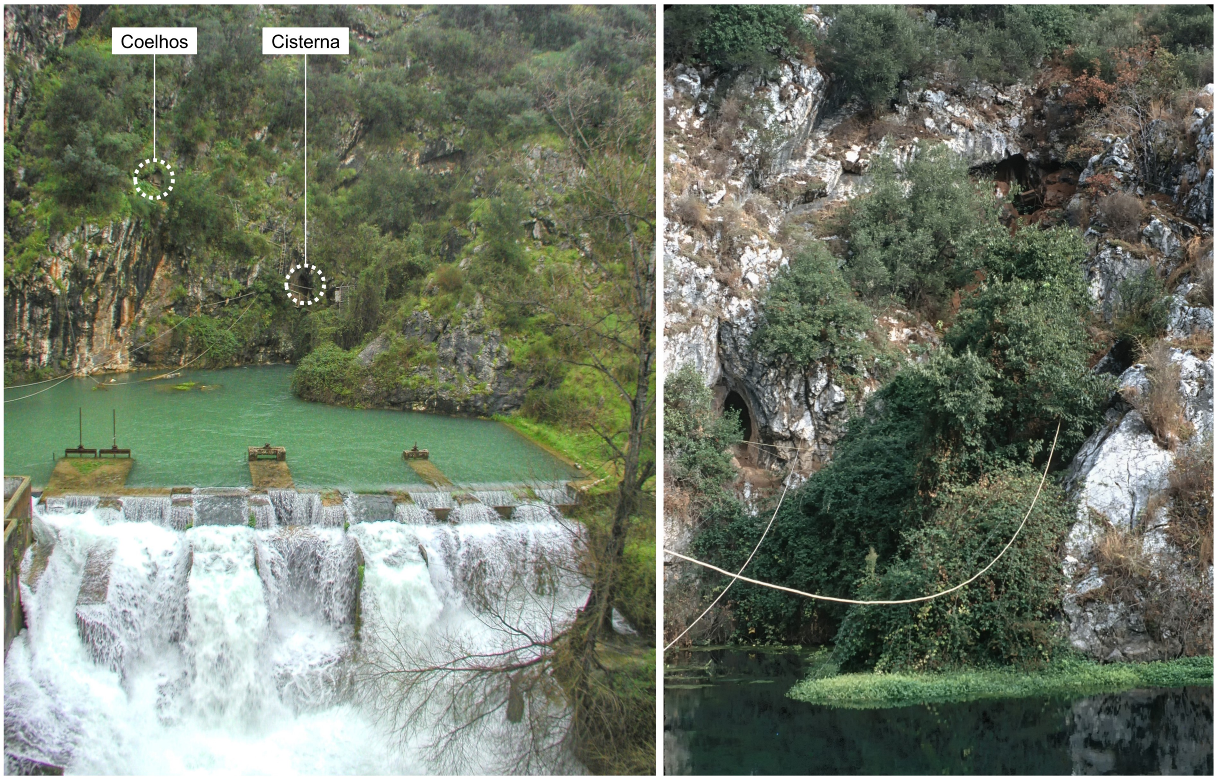

Fig. S4. The Almonda spring in winter (left) and summer (right). Relative to Galeria da Cisterna, Lapa dos Coelhos is located *c.* 15 m to south and *c.* 10 m higher up in the scarp face.

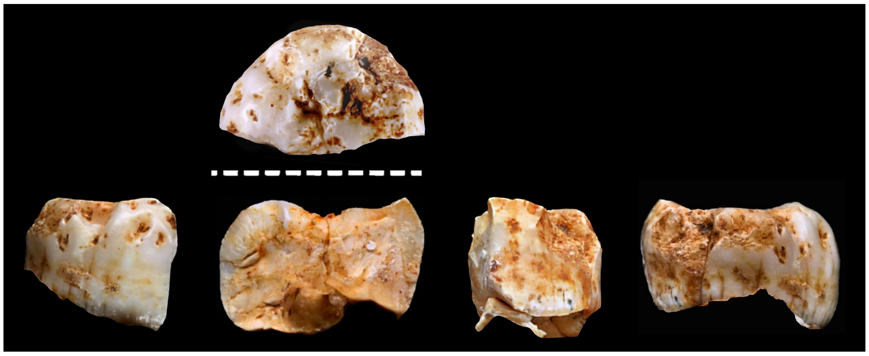

Fig. S5. The Oliveira 8 right molar (M_2 or M_3) (OLV-N16-373).

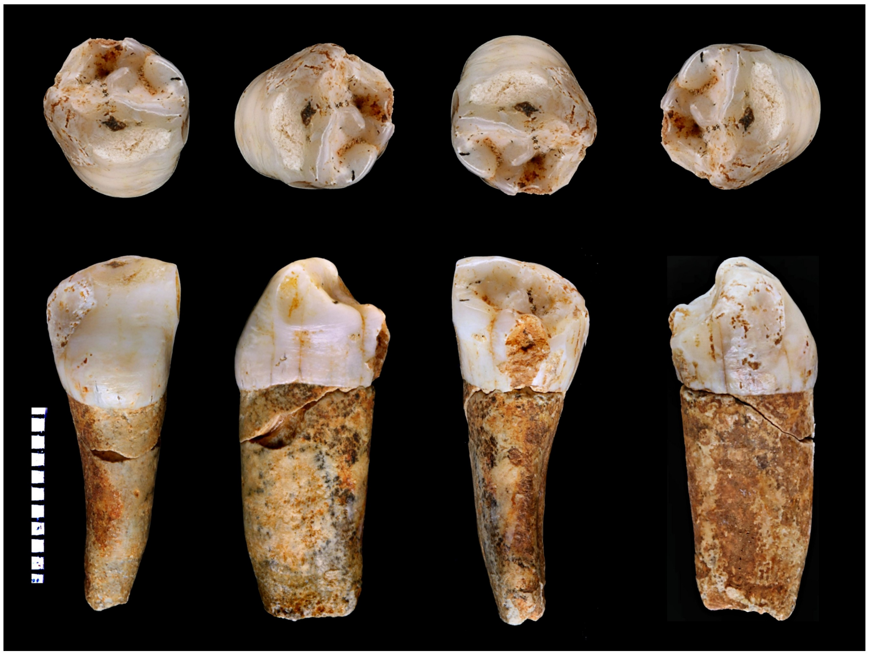

Fig. S6. The Oliveira 9 right P_3) (OLV-N15-383).

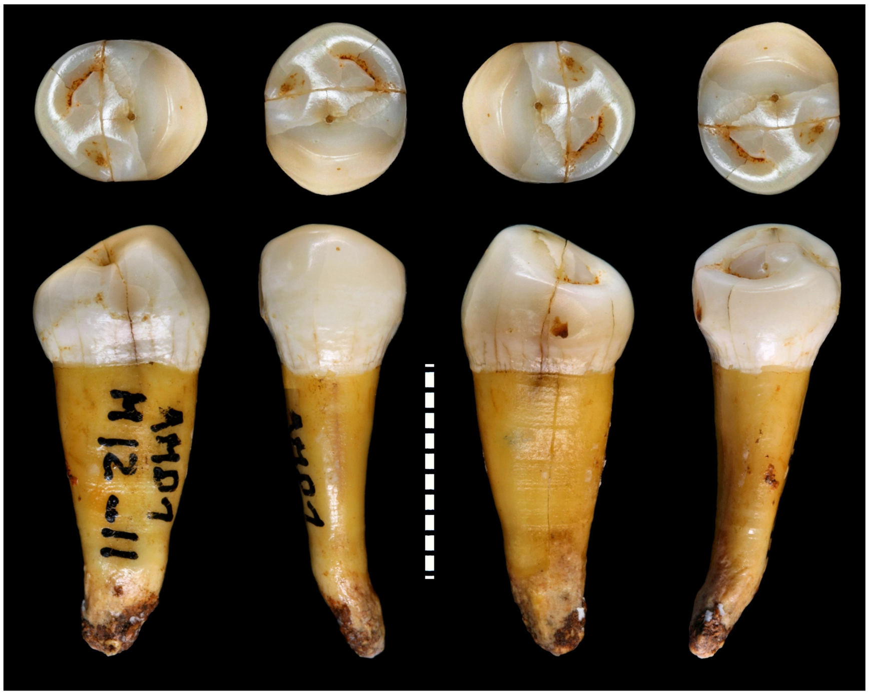

Fig. S7. The Cisterna 2 left P_3 (AMD1-M12-11).

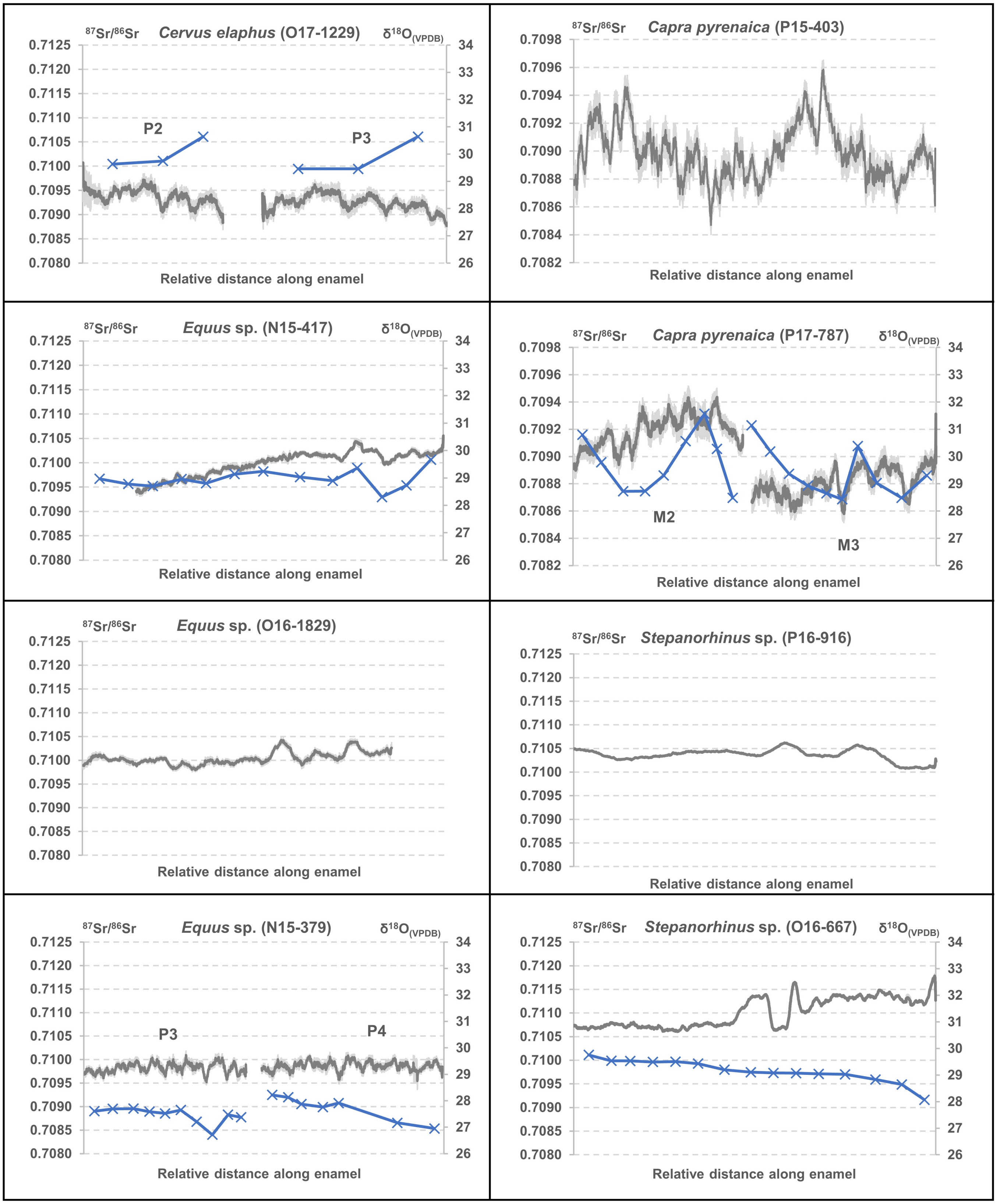

Fig. S8. Isotope data (strontium and oxygen) for the analysed Oliveira faunal remains, plotted from left (top of crown) to right (enamel cervix), following the growth axis..

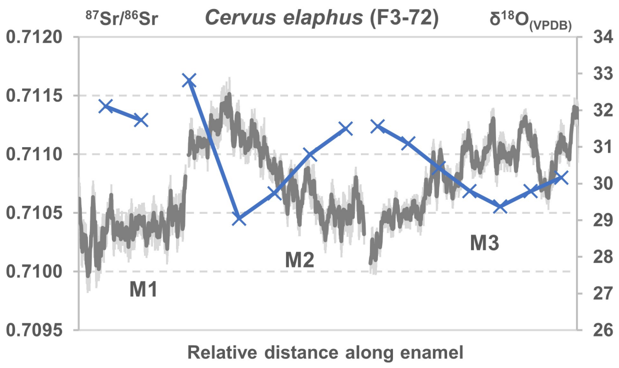

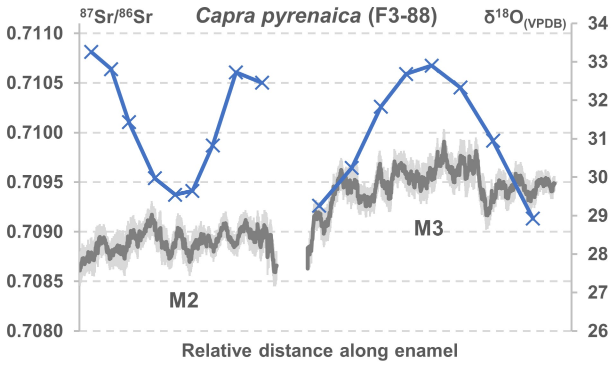

Fig. S9. Isotope data (Sr and O) for the analysed Cisterna faunal remains.

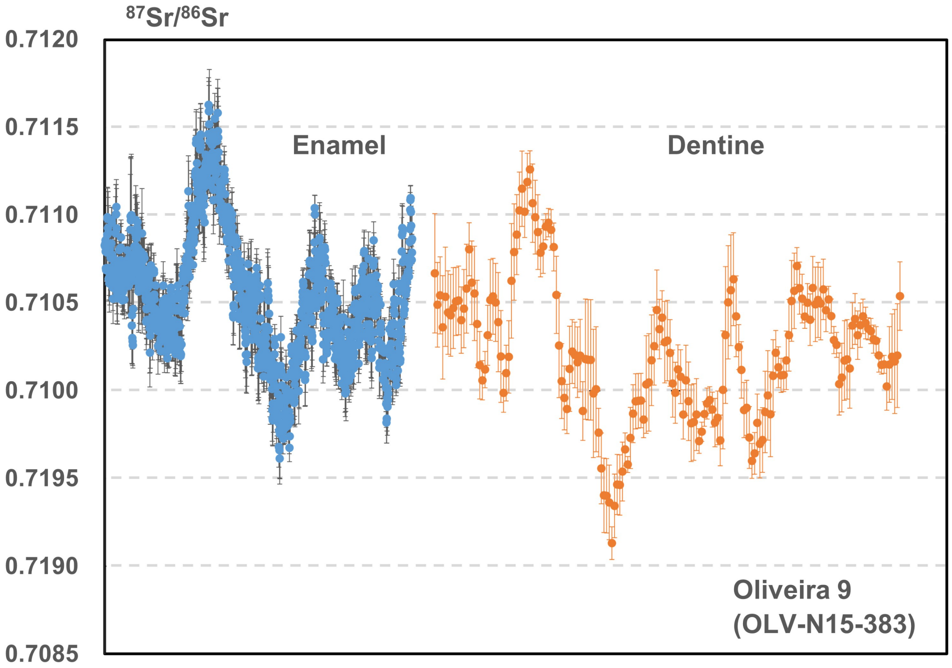

Fig. S10. Sr isotope values along the enamel and dentine for Oliveira 9. Note that, because of the geometry of tooth mineralization and the positioning of the analyses, the enamel and dentine tracks do not represent the same timescales. For easier comparison between the enamel and dentine, the data is plotted from the crown (left) towards the root (right). whilst human enamel and dentine mineralisation rates are yet to be fully understood, the plotted section of the enamel profile appears to reflect the same biogenic fluctuations in 87Sr/86Sr as the dentine - the enamel profile has been truncated for easier comparison.

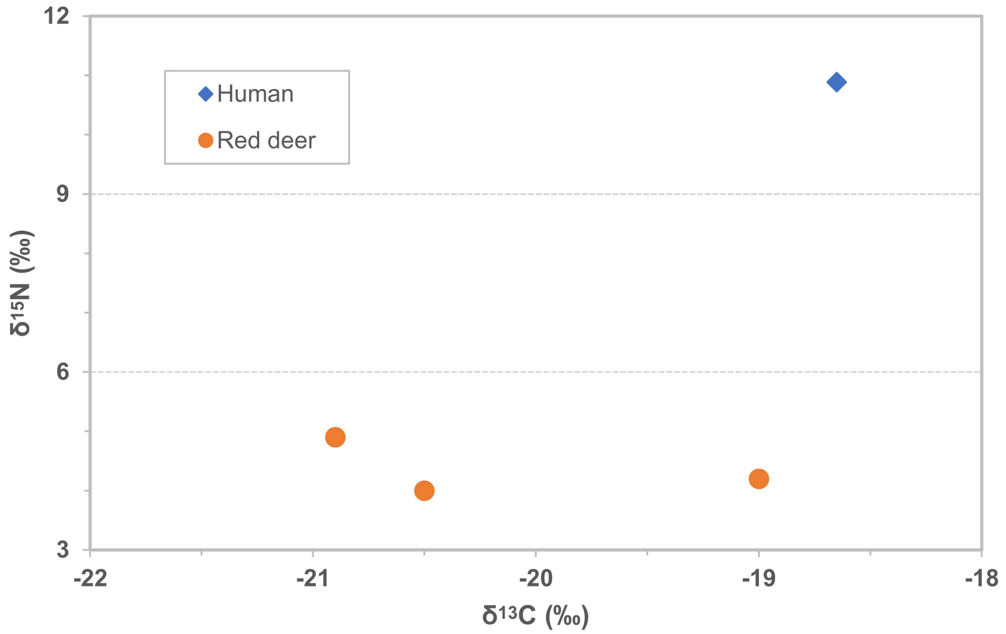

Fig. S11. Stable carbon and nitrogen isotope plot for the Upper Palaeolithic human from Galeria da Cisterna and Upper Palaeolithic red deer from Lapa dos Coelhos.

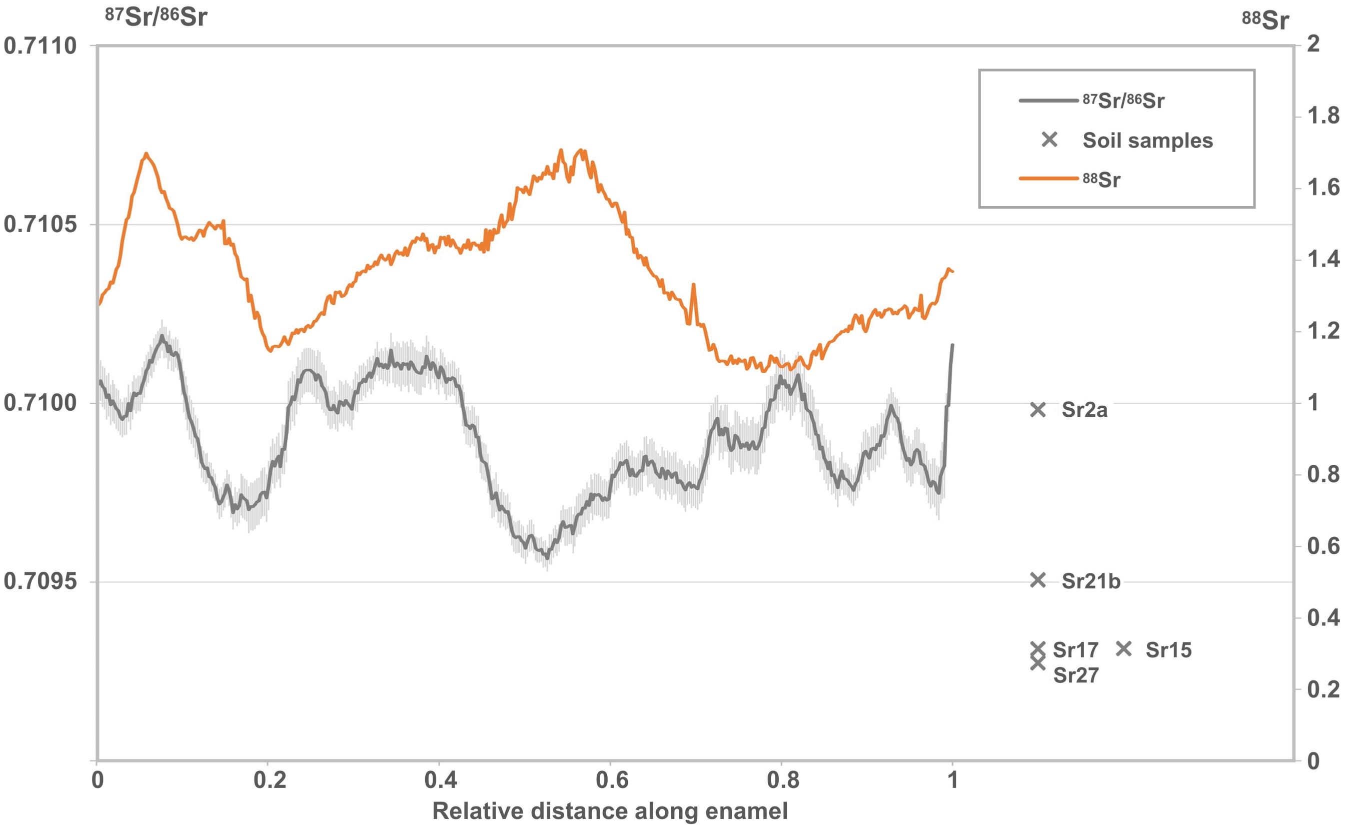

Fig. S12. Variation in ^{88}Sr across the growth axis of the tooth (orange line) plotted against ^{87}Sr/^{86}Sr for the Upper Palaeolithic Galeria da Cisterna human. ^{88}Sr is considered to be a proxy for strontium concentration..

Supplementary Tables

Table S1. Samples analysed in this study.

Sample ID	Element	Taxon	Layer	Isotopes
**Gruta da Oliveira (Mousterian)**				
N16-373 (Olv8)	Lower molar	Homo (Neandertal)	22	Sr
N15-383 (Olv9)	Lower first premolar	Homo (Neandertal)	22	Sr
N15-417	Mandible fragment with molar	*Equus* sp.	22	Sr, O
N15-379	Mandible fragment with dental arcade	*Equus* sp.	22	Sr, O
O16-1829	Molar fragment	*Equus* sp.	24	Sr
P16-787	Mandible with two molars	*Capra pyrenaica*	20	Sr, O
P15-403	Molar	*Capra* pyrenaica	21	Sr
O17-1229	Maxillary fragment with dental arcade	*Cervus elaphus*	20	Sr, O
P16-916	Molar fragment	*Stephanorhinus* sp.	26	Sr
O15-667	Molar fragment	*Stephanorhinus* sp.	21	Sr, O
**Galeria da Cisterna (Magdalenian)**				
AMD1-M12-11	Lower left premolar	*Homo* sp.	3	C, N, S, Sr
**Lapa dos Coelhos (Magdalenian)**				
CLH-F3-88	Maxilla with dental arcade	*Capra pyrenaica*	4	C, N, S, Sr
CLH-F3-72	Maxilla with dental arcade	*Cervus elaphus*	4	C, N, S, Sr
CLH-F3-73	Distal metacarpal	*Cervus elaphus*		C, N

Table S2. Radiocarbon dated samples from Galeria da Cisterna and Lapa dos Coelhos.

Sample ID	Taxon	Element	Layer	Lab Code
**Galeria da Cisterna**				
AMD1-M12-16	Large mammal	Long bone fragment	3	GrA-9722
AMD1-M12-11	*Homo* sp.	Lower left premolar	3	OxA-41483
AMD1-M11-100	*Capra pyrenaica*	Phalange	3	OxA-11129
**Lapa dos Coelhos**				
CLH-G2-35	Large mammal	Long bone fragment	3	OxA-41487
CLH-G2-8	*Cervus elaphus (?)*	Long bone fragment	3	OxA-41486
–	*Cervus elaphus*	Patella	3	GrA-18376
CLH-F3-73	*Cervus elaphus*	Distal metacarpal	4	OxA-41485
CLH-F3-72	*Cervus elaphus*	Mandible with P_2 - M_3	4	OxA-41484
–	*Pinus sylvestris*	Charcoal	4	GrA-18377

Table S3. Ages and chemistry data for the radiocarbon dated samples from Galeria da Cisterna and Lapa dos Coelhos. Dates calibrated using OxCal 4.4 and IntCal 20 (13).

Lab Code	Pretreatment (a)	%C	C:N	Age	cal BP (2σ)
**Galeria da Cisterna**					
GrA-9722	–			10820 ± 60	12680 - 12880
OxA-41483	AF	42.8	3.2	11122 ± 34	12925 - 13108
OxA-11129	AF*	41.9	3.5	11775 ± 80	13500 - 13820
**Lapa dos Coelhos**					
OxA-41487	AF	42.9	3.2	11629 ± 39	13363 - 13593
OxA-41486	AF	43.4	3.3	11649 ± 38	13440 - 13596
GrA-18376	–	–	–	11660 ± 60	13354 - 13734
OxA-41485	AF	41.6	3.4	12038 ± 45	13800 - 14038
OxA-41484	AF	41.4	3.3	12130 ± 43	13811 - 14117
GrA-18377	–	–	–	12240 ± 60	14362 - 14804

(a) AF = ABA collagen extraction and ultrafiltration. AF* = solvent wash (acetone, methanol, chloroform) followed by ABA collagen extraction and ultrafiltration. Full descriptions of the Oxford Radiocarbon Accelerator Unit pretreatment methods are available in Brock et al. (14).

Table S4. Sr isotope values for soil leachates.

Ref	Location	Geology	Lat/Long	$^{87}Sr/^{86}Sr$
**Sr1a**	Riachos	Just above floodplain	39°27'14.26"N 8°31'11.24"W	0.713034 ± 0.000022
**Sr2a**	Chamusca	Edge of Tagus floodplain	39°22'15.53"N 8°28'3.00"W	0.709982 ± 0.000012
**Sr3a**	Alpiarça	First terrace above floodplain: area probably still floods	39°18'16.42"N 8°29'57.66"W	0.715395 ± 0.000015
**Sr4**	Balsas	Hills above floodplain: Oligocene or Pliocene gravels	39°19'18.26"N 8°22'56.60"W	0.71634 ± 0.000017
**Sr5**	Entroncamento	Quaternary terrace of the Tagus: gravel and sand	39°28'36.52"N 8°27'32.69"W	0.717235 ± 0.000016
**Sr6**	Chão da Telha	Cambrian schist/slate	39°45'10.04"N 8° 7'31.87"W	0.716529 ± 0.000016
**Sr7**	Ferreira de Zêzere	Pliocene sand & gravel	39°42'13.50"N 8°18'30.85"W	0.716375 ± 0.000017
**Sr8**	Ceras	Mesozoic interstratified limestone and marl	39°41'40.09"N 8°21'9.43"W	0.713158 ± 0.000014
**Sr9**	Pedreira	Miocene (thick) sand & gravel on top of bedrock	39°38'38.29"N 8°25'39.18"W	0.714017 ± 0.00002
**Sr10**	Ervideiras	Palaeozoic metamorphic rock containing mica and quartz	39°33'2.88"N 8°22'1.56"W	0.716476 ± 0.000016
**Sr11**	Pedreira do Galinha	Jurassic limestone	39°34'21.47"N 8°35'10.97"W	0.708754 ± 0.000015
**Sr12**	Fátima	Jurassic limestone with clayey terra rossa soils (deeper than at Sr11)	39°36'57.31"N 8°39'1.22"W	0.708099 ± 0.000014
**Sr13**	Caxarias	Sand with gravel	39°41'46.97"N 8°32'14.46"W	0.713363 ± 0.000013
**Sr14**	Alvados	Bottom of Polje of Alvados	39°33'37.37"N 8°47'4.74"W	0.708807 ± 0.000013
**Sr15**	Vale da Serra	Red soils (local name: "felgar") in field of synclinal valley	39°30'11.63"N 8°38'59.93"W	0.709314 ± 0.000013
**Sr16**	Salir de Matos	Colluvial sands over Upper Jurassic sandstone bedrock	39°26'2.72"N 9° 6'11.92"W	0.713878 ± 0.000017
**Sr17**	Nadadouro	Pliocene sands	39°25'52.14"N 9°11'11.65"W	0.709314 ± 0.000013
**Sr18**	Azinheira	Sandy Cenozoic soils on Tertiary sandstone	39°20'1.10"N 8°55'0.84"W	0.713724 ± 0.000017
**Sr19**	Torre de Bispo	Not recorded	39°21'23.03"N 8°39'26.89"W	0.711347 ± 0.000014
**Sr21b**	Almonda	Miocene immediately downstream from Almonda.	39°29'52.48"N 8°36'34.85"W	0.709507 ± 0.000015
**Sr22**	Castelo de Vide	Granite	39°26'6.72"N 7°31'1.60"W	0.736187 ± 0.000016
**Sr23**	Pombeira	Ordovician schist/philite	39°41'47.36"N 8°14'3.77"W	0.719667 ± 0.000013
**Sr24**	Portela de Nexebra	Triassic quartz sandstone	39°41'24.29"N 8°20'21.84"W	0.716687 ± 0.000016
**Sr25**	Terras Pretas	Middle to Late Miocene coarse sand & gravel	39°32'5.06"N 8°31'10.63"W	0.711166 ± 0.000016
**Sr26**	Santo Amaro (Ourém)	Gravel with sand cover at high elevation	39°37'50.84"N 8°35'13.34"W	0.716868 ± 0.000015
**Sr27**	Nazaré (coast)	Pleistocene sand dune with some Holocene reworking	39°36'52.92"N 9° 4'59.66"W	0.709275 ± 0.000018
**Sr28**	Arruda dos Pisões	Chromic cambisoil on Middle to Late Miocene coarse sand & gravel bedrock	39°19'12.2" N 8°50'19.2" W	0.711291 ± 0.000016

Table S5. Stable carbon and nitrogen isotope data for the Upper Palaeolithic human from Galeria da Cisterna and Upper Palaeolithic red deer from Lapa dos Coelhos.

Sample ID	Description	Lab #	Age BP	$\delta^{13}C$ (‰)[1]	$\delta^{15}N$ (‰)[2]	C:N	Reference
**Galeria da Cisterna**							
AMD1-M12-11	human (tooth root)	OxA-41483	11122 ± 34	-18.65	10.89	3.3	This study
**Lapa dos Coelhos**							
CLH-F3-72	red deer	OxA-41484	12130 ± 43	-20.5	4.0	3.4	This study
CLH-F3-73	red deer	OxA-41485	12038 ± 45	-20.9	4.9	3.3	This study
CLH-G2-8	red deer	OxA-41486	11649 ± 38	-19.0	4.2	3.2	This study

[1] Values relative to vPDB (standard error: 0.23)
[2] Values relative to AIR (standard error: 0.20)
